# Programmatic assessment and competency development in postgraduate medical education: a systematic review and narrative synthesis

**DOI:** 10.3389/fmed.2026.1873126

**Published:** 2026-07-16

**Authors:** İbrahim Ulaş Özturan, Pınar Daylan, Cansu Alyeşil Özturan, Dilek Kitapçıoğlu, Levent Altıntaş

**Affiliations:** 1 Department of Medical Education, Acıbadem University, İstanbul, Türkiye; 2 Department of Emergency Medicine, Kocaeli University, Kocaeli, Türkiye; 3 Department of Medical Education, Kocaeli University, Kocaeli, Türkiye; 4 Kocaeli City Hospital, Ministry of Health, Kocaeli, Türkiye

**Keywords:** clinical competence, competency-based medical education, narrative synthesis, postgraduate medical education, programmatic assessment, systematic review, workplace-based assessment

## Abstract

**Introduction:**

Programmatic assessment (PA) is widely endorsed as a way to organize assessment around competency development in postgraduate medical education (PGME), yet no systematic review has synthesized the empirical evidence in this setting. We therefore synthesized the evidence on its contribution to competency development in PGME and on the factors that shape its implementation.

**Methods:**

A systematic review was conducted following PRISMA 2020 guidelines (PROSPERO: CRD420261349850). We searched MEDLINE, Embase, Scopus, Web of Science, and EBSCOhost from January 2005 onward. Each included study was mapped against four operational features of PA derived from van der Vleuten et al. Methodological quality was assessed using the Mixed Methods Appraisal Tool (MMAT), and findings were synthesized using a narrative approach.

**Results:**

Twenty studies met inclusion criteria. Four reported competency-related outcomes, all with observational designs. Two evaluated purposefully designed PA systems, and two analyzed milestone-based data generated within regulated competency-based training without an explicitly designed PA system. These studies reported increases in competency ratings and improved examination outcomes over training, though none could establish a causal link to the assessment system. The remaining 16 studies focused on implementation processes, stakeholder perceptions, and measurement properties. Challenges including feedback documentation barriers, misalignment between intended and perceived assessment stakes, and variable stakeholder engagement were reported consistently across settings. Methodological quality was uneven across the included studies (nine rated High, six Moderate, and five Low on a descriptive MMAT-based quality index).

**Discussion:**

The empirical evidence for PA in PGME remains limited relative to its theoretical foundations. Most evidence reflects how stakeholders experience the system or whether assessment tools produce reliable data, rather than whether the system improves competency or patient care. Future research should extend outcome measurement beyond system-internal metrics and account for the contextual specificity of PA across diverse educational and regulatory environments.

## 1 Introduction

Competency-based medical education (CBME) has fundamentally reshaped the structure of postgraduate medical education (PGME), shifting the focus from time-based progression to the demonstration of defined competencies required for independent practice (1–3). Central to this shift is the recognition that assessment must move beyond summative, point-in-time evaluations toward systems capable of capturing the longitudinal development of competence across multiple domains and contexts (4), building on the educational use of workplace-based assessment (WBA) (5).

Responding to this need, van der Vleuten et al. (6) formalized programmatic assessment (PA), defining it as a purposefully designed system in which multiple low-stakes data points are longitudinally aggregated, triangulated across modalities, used principally for learner-directed feedback, and synthesized by a competence committee for high-stakes progression decisions across a continuum of stakes (6, 7). This operationalizes the distinction between assessment *for* learning and assessment *of* learning articulated by Schuwirth and van der Vleuten (7): low-stakes data serve as formative inputs whose aggregation supports defensible high-stakes decisions. PA is best understood as one operationalization of CBME’s assessment requirements — not as synonymous with CBME itself — with theoretical roots predating the international CBME movement (8). These roots include early work on qualitative, portfolio-based judgment as an alternative to purely psychometric evaluation in clinical training (9).

Over the past decade, PA has been incorporated into PGME systems internationally, embedded by national accreditation and training bodies in specialty training programs (10). The conceptual foundations and design principles have been extensively described (7, 8, 11, 12), and formal consensus on principles has been articulated (13, 14). A growing number of empirical studies have examined how PA functions in practice, reporting on stakeholder perspectives, implementation processes, feasibility, assessment quality, and outcomes. Empirical work in undergraduate medical education has documented impacts on learner approaches (15), but evidence specific to PGME has not been systematically synthesized, and findings remain fragmented across disciplines, countries, and research traditions.

The purpose of this systematic review is to identify, appraise, and narratively synthesize original empirical studies on PA in PGME, with particular attention to evidence on competency development outcomes, implementation processes, stakeholder experiences, assessment quality, and feasibility across training contexts.

## 2 Methods

### 2.1 Protocol and registration

This systematic review was registered prospectively with PROSPERO (CRD420261349850) and conducted in accordance with the Preferred Reporting Items for Systematic Reviews and Meta-Analyses (PRISMA) 2020 guidelines. The review protocol specified the research question, eligibility criteria, search strategy, data extraction procedures, and synthesis approach prior to study selection.

### 2.2 Research questions and PICOS framework

The primary research question was: What is the effectiveness of PA in improving competency development in PGME? Two subquestions guided the synthesis: (a) What are learners’ and faculty members’ perceptions of PA in this context? and (b) What challenges and facilitators are reported in implementing PA? The review was structured around a PICOS framework. Population: residents, fellows, or other postgraduate trainees in any medical specialty and geographic region. Intervention: PA or equivalent concepts (e.g., “systems of assessment,” “assessment system”) implemented as part of postgraduate training. Comparison: traditional assessment methods, other forms of assessment, or no formal comparison where studies reported pre-post or single-arm outcomes. Outcomes: competence development, feedback quality, assessment utility, learner progression, faculty satisfaction, or implementation outcomes. Study design: quantitative, qualitative, and mixed-methods empirical studies. Systematic reviews were retrieved and screened for citation chaining but were not themselves eligible as evidence sources; evaluation reports were eligible only where they reported original empirical data.

### 2.3 Eligibility criteria

Based on the PICOS framework above, studies were included if they involved postgraduate medical trainees, described or evaluated a programmatic or system-based approach to assessment consistent with the framework described by van der Vleuten et al. (6), employed an empirical research design, and reported on at least one of the specified outcome domains. Only English-language publications from January 2005 onward were considered. Studies were excluded if they focused exclusively on undergraduate medical education, nursing, or allied health professions without a postgraduate medicine component; were conference abstracts, editorials, commentaries, letters, or opinion pieces without original research data; or did not clearly describe or evaluate a PA system.

To make the boundary between PA and CBME more broadly verifiable, a PA system was operationally defined by four features derived from van der Vleuten et al. (6): (a) purposeful longitudinal aggregation of multiple low-stakes data points; (b) triangulation across assessment modalities; (c) a formative-first orientation in which individual data points are used principally for feedback; and (d) committee-based synthesis of aggregated data for high-stakes decisions. Each included study was mapped against these four features, and the resulting fidelity matrix is reported in Supplementary Material 5. Studies that analyzed system-internal milestone data generated within regulated competency-based training, without describing a purposefully designed PA system meeting all four features, were retained but analyzed as a distinct subgroup (milestone-based CBME without explicit PA design) rather than as direct evaluations of PA.

The same operational definition governed the boundary between systems and their component tools. Studies developing or validating a single enabling tool — for example, a mobile application to facilitate assessment delivery (16) or a natural-language-processing model to organize narrative comments (17) — were excluded as tool-development studies, whereas studies empirically evaluating a deployed multi-component system of assessment (e.g., the piloted system of entrustable professional activity (EPA), procedural, nontechnical, and examination-based assessments in Woodworth et al. (18)) were included. The reasons for each full-text exclusion are listed in Supplementary Material 2.

### 2.4 Information sources and search strategy

We searched five databases: MEDLINE (Ovid), Embase (Ovid), Scopus, Web of Science, and EBSCOhost, the last of which included Academic Search Ultimate, CINAHL Complete, ERIC, and Library, Information Science and Technology Abstracts. The search strategy combined terms related to four core concepts: PA and related assessment system terminology, PGME, competency and learning outcome measures, and publication date. Search strategies were adapted for each database; for example, the Web of Science strategy incorporated proximity operators and additional terms such as “programmatic feedback” and “assessment program*” to account for variation in indexing. The full search strategy for each database is provided in Supplementary Material 1.

### 2.5 Study selection

**Figure 1:**
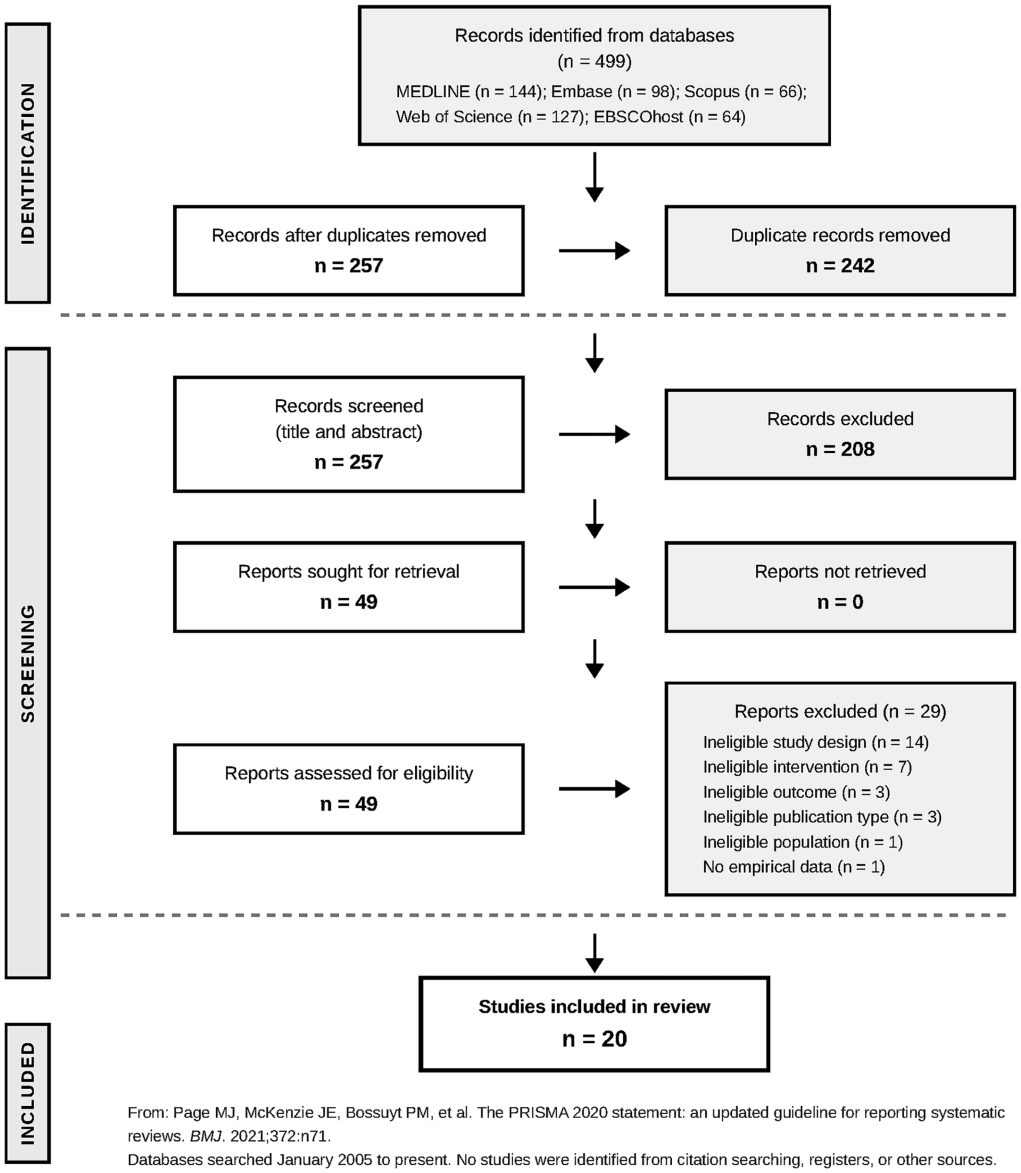
PRISMA 2020 flow diagram.

We conducted study selection in two phases. In the first phase, two reviewers independently screened the titles and abstracts of all identified records against the eligibility criteria. In the second phase, full texts of potentially eligible studies were retrieved and assessed for inclusion. At each phase, a calibration exercise was conducted on an initial subset of records to ensure consistent application of the criteria. Disagreements were resolved through discussion and, where necessary, consultation with a third reviewer. The database search identified 499 records (MEDLINE: 144; Embase: 98; Web of Science: 127; Scopus: 66; EBSCOhost: 64). After removal of 242 duplicates, 257 unique records were screened at the title and abstract level. Of these, 49 proceeded to full-text assessment, and 20 studies met all inclusion criteria (Figure 1). This left 208 records excluded at title/abstract screening. The 29 excluded full-text articles, with reasons for exclusion, are listed in Supplementary Material 2.

### 2.6 Data extraction

Data were extracted using a standardized form comprising 25 fields organized across five domains: study identification (study ID, authors, year, country, journal), design and methods (study design, data collection, data analysis), population (specialty, training level, sample size, participant characteristics), PA characteristics (terminology, components and tools, feedback and decision-making processes, implementation duration, comparator), and outcomes and findings (primary outcome, competency domain, key quantitative and qualitative findings, secondary findings, direction of effect). Two reviewers independently extracted data from each included study using the standardized form, and a third reviewer adjudicated any discrepancies. Where studies reported effect estimates, measures of precision (95% confidence intervals) were extracted when available; their absence in a source is reported as such.

### 2.7 Quality appraisal

Methodological quality was appraised using the Mixed Methods Appraisal Tool (MMAT) version 2018 (19). Each study was classified into one of the five MMAT categories — qualitative, quantitative randomized, quantitative non-randomized, quantitative descriptive, or mixed methods — and rated on two screening items and five category-specific criteria, each scored “Yes,” “No,” or “Cannot tell.” Because the MMAT developers explicitly discourage the calculation of summary quality scores and recommend reporting item-level ratings (19), the count of “Yes” ratings on the five category-specific criteria (Met/5) is reported solely as a *descriptive quality index* to support transparent communication, not as a validated quality score; item-level ratings for every study are reported in full in Supplementary Material 4, and recurring item-level limitations are considered alongside the synthesized findings in the Results. Descriptive quality ratings were defined *a priori* as High (Met/5 ≥ 4), Moderate (Met/5 = 3), and Low (Met/5 ≤ 2). Two reviewers independently scored every study against the seven MMAT items, documenting each rating with a direct quotation and page reference from the source paper. A third reviewer resolved all disagreements by returning to the source paper and recording the quoted evidence supporting each final rating. Pre-adjudication item-level agreement was 86.7% (Cohen’s *κ* = 0.707, substantial agreement (20)). Per-study scorecards, the disagreement log, and inter-rater agreement details are provided in Supplementary Material 4.

### 2.8 Synthesis approach

Given the heterogeneity in study designs, populations, assessment systems, and outcome measures, a narrative synthesis was conducted following the framework described by Popay et al. (21). Meta-analysis was not feasible due to the diversity of study designs and the absence of comparable quantitative effect estimates across studies. The synthesis was organized into two strands: (1) evidence relating to competency development outcomes, and (2) evidence relating to implementation processes, stakeholder experiences, and psychometric properties of PA systems. Within each strand, studies were grouped thematically, and the direction and strength of findings were assessed in relation to study quality.

For studies measuring competency development using ratings generated by the PA system itself (system-internal metrics), the review flags the risk of construct circularity, whereby the assessment system both produces the data and defines the outcome, precluding independent validation of competency gains. This validity threat was anticipated to affect the interpretation of competency-development evidence, particularly for studies using milestone ratings as both process and outcome, and it is applied explicitly in Section 3.2.

At the interpretation stage, outcome evidence was descriptively mapped to the four levels of the New World Kirkpatrick Model (22) as a non-pre-specified interpretive lens, used to show where empirical attention has concentrated rather than to rank evidence quality (23). Implementation-stage evidence was additionally characterized using Proctor et al.’s implementation-outcomes taxonomy (24). Moore et al.’s expanded outcomes framework (25) is noted in the Discussion as a contextually tailored alternative for future work.

### 2.9 Certainty assessment

Confidence in the principal qualitative and implementation findings of the synthesis was assessed using the GRADE-CERQual approach (26), which evaluates each synthesized finding across four components: methodological limitations of the contributing studies (informed by the MMAT item-level ratings), coherence, adequacy of data, and relevance to the review question. The resulting Summary of Qualitative Findings, with the assessed confidence level and rationale for each finding, is presented in Supplementary Material 6. GRADE was not applied to the quantitative strand because the included studies comprised heterogeneous, predominantly descriptive designs without pooled or comparable effect estimates; certainty in quantitative findings is therefore discussed narratively in relation to design limitations and risk of bias.

## 3 Results

### 3.1 Study characteristics, PA Fidelity, and quality appraisal

The 20 included studies were published between 2014 and 2025, with 12 (60%) appearing from 2020 onward. Most originated from Canada (n = 9) and the United States (n = 7), with single studies from Australia and Ireland and two multinational studies. Emergency medicine was the most frequently represented specialty (*n* = 6), followed by family medicine (*n* = 5), internal medicine (*n* = 3), surgery (*n* = 2), and single studies in obstetrics and gynecology and anesthesiology; two studies involved multiple specialties. Study designs included qualitative (*n* = 6), quantitative descriptive (*n* = 9), quantitative non-randomized (*n* = 2), and mixed methods (*n* = 3); none employed a randomized controlled design. Sample sizes, expressed as the number of participants (trainees, faculty, or program members), ranged from 5 to 3,872 (the lower bound reflects the auto-ethnographic researcher cohort in Paternotte et al. (27), rather than enrolled trainees); several studies additionally reported larger data units, such as assessment records, evaluations, or fieldnotes, rather than unique individuals (see Table 1 for detailed sample descriptors).

**Table 1:** Characteristics and methodological quality appraisal of the 20 included studies.

First author, year	Country	Specialty	Study design (MMAT category)	Sample	PA System	Primary outcome focus	MMAT index (Met/5)
Acai et al. (39)	Canada	EM	Qualitative (1)	16 faculty	McMAP	Stakeholder perceptions	High (5)
Ashmanet al (32).	Australia	Orthopedic surgery	Mixed methods (5)	571 trainees; 46,180 WBAs; survey 322 of 1,778 invited (18%)	AOA21 CBT	Competency + implementation	High (4)
Caretta-Weyer et al. (41)	United States	EM	Quant desc (4)	8 programs; 391 residents	EPA-based CBME	Feasibility, acceptability	Moderate (3)
Chan et al. (33)	Canada	EM	Quant non-rand (3)	15 residents; 50 reports (25 pre, 25 post)	McMAP	Competency (report quality)	Moderate (3)
Gauthier et al. (38)	Canada	IM	Qualitative (1)	20 residents	EPA-based WBA (CBD)	Assessment-seeking behavior	High (5)
Hauff et al. (30) ‡	United States	EM	Quant desc (4)	28 interns	Multisource milestone	Baseline competency measurement	High (4)
Lee and Ross (50)	Canada	FM	Quant non-rand (3)	6,863 fieldnotes	CBAS (e-portfolio)	Self-preceptor congruence	Moderate (3)
McEwen et al. (44)	Canada	FM	Mixed methods (5)	~130 residents	PASS portfolio	Implementation feasibility	Low (0)
O’Keeffe et al. (48)	Ireland	Surgery	Quant desc (4)	114 trainees	CTAS	Measurement validity	High (5)
Park et al. (46)	United States	IM	Quant desc (4)	142 residents; 2,701 evals	NAS milestone evaluations	Measurement validity	Moderate (3)
Park et al. (28)†	United States	IM	Quant desc (4)	34 residents	Scoring Grid Model (milestone reporting)	Competency + validity	Moderate (3)
Park et al. (29)†	United States	FM	Quant desc (4)	3,872 residents; 514 programs	ACGME FM Milestones	Competency + validity	High (5)
Paternotte et al. (27)	Canada/Netherlands	OBGYN	Qualitative (1)	5 researchers + informants	CBD / LOGO	Cross-system comparison	Low (1)
Perry et al. (43)	United States	EM	Quant desc (4)	~64 residents	Master Assessment Plan	Implementation feasibility	Low (0)
Rich et al. (40)	Canada	EM	Qualitative (1)	22 participants	CBD with EPAs	Operationalization challenges	High (5)
Rich et al. (45)	Canada	Multiple	Qualitative (1)	17 CC/AA; 4 programs	CBD model	Committee functioning	High (5)
Ross et al. (51)	Canada	FM	Mixed methods (5)	17 programs; ~3,003 grads	CRAFT	Meta-evaluation	Low (2)
Schultz and Griffiths (42)	Canada	FM	Quant desc (4)	~150 residents (2013–2015)	EPA-based CBME (36 EPAs)	Implementation lessons	Low (0)
Schut et al. (37)	International	Medicine (mixed)	Qualitative (1)	26 learners (11 postgraduate); 5 programs	PA with portfolios	Stakeholder perceptions	High (5)
Woodworth et al. (18)	United States	Anesthesiology	Quant desc (4)	490 residents; 7 programs	EPA + procedural + NTSA + OSCE	Measurement validity	Moderate (3)

MMAT category shown in parentheses: 1, qualitative; 3, quantitative non-randomized; 4, quantitative descriptive; 5, mixed methods. Met/5, number of category-specific MMAT criteria met (High ≥ 4; Moderate = 3; Low ≤ 2). † Milestone-based CBME subgroup; ‡ single-time-point assessment (modality evidence). Full appraisal in Supplementary Material 4; programmatic-assessment fidelity mapping in Supplementary Material 5.

Mapping each study against the four operational features of PA (Section 2.3; Supplementary Material 5) showed that 17 studies evaluated systems designed to embody all or most of these features. Two studies — Park et al. (28) and Park et al. (29) — analyzed milestone rating data generated within accreditation-mandated competency-based training, in which committee-based synthesis and longitudinal data accumulation are present as regulatory requirements but a purposefully designed PA system, in the sense of van der Vleuten et al. (6), is not described; these are treated throughout as a distinct subgroup (milestone-based CBME without explicit PA design). One further study, Hauff et al. (30), applied a multisource, multi-station assessment at a single time point (intern orientation) rather than as a longitudinal system, and its findings are interpreted as evidence about assessment-modality properties rather than about PA as a longitudinal system.

Methodological quality varied across the included studies. On the descriptive quality index (Section 2.7), Met/5 values ranged from 0 to 5. Nine studies (45%) were classified as High quality (Met/5 ≥ 4), six (30%) as Moderate (Met/5 = 3), and five (25%) as Low quality (Met/5 ≤ 2). The five Low-quality studies were predominantly innovation reports, brief program descriptions, or auto-ethnographic accounts in which limited methodological reporting (sampling frame, response rate, analytic procedure, or integration of mixed-methods strands) constrained MMAT scoring rather than any inherent flaw of the underlying programs being described. Auto-ethnography is a recognized qualitative research tradition (31); the lower MMAT scores for studies in this category reflect how the report’s reporting maps to MMAT criteria rather than judgments about the legitimacy of the underlying methodology. Recurring item-level limitations across the included studies were sample representativeness (items 4.2 and 1.4), nonresponse bias (item 4.4), and integration of qualitative and quantitative components in mixed-methods designs (items 5.2–5.5); where findings below rest on studies affected by these limitations, the quality rating is noted at the point of citation. Pre-adjudication inter-rater agreement was 86.7% at the item level (Cohen’s *κ* = 0.707); 19 disagreements were resolved by third-reviewer adjudication, including one disagreement on MMAT category assignment. The characteristics and quality appraisal of all included studies are presented in Table 1, and Table 2 maps each study to the thematic findings of the synthesis, with the direction of findings and quality rating.

**Table 2:** Synthesis matrix: study contributions to thematic findings, with direction and quality rating.

Study	Quality rating (Met/5)	Competency outcomes	Engagement and culture	Feasibility and resources	Decision-making and problem identification	Measurement properties	Contextual variation
Acai et al. (39)	High (5)	—	M	—	—	—	—
Ashmanet al (32).	High (4)	M	C	M	—	—	—
Caretta-Weyer et al. (41)	Moderate (3)	—	C	M	—	—	—
Chan et al. (33)	Moderate (3)	S	—	—	—	M	—
Gauthier et al. (38)	High (5)	—	C	—	—	—	—
Hauff et al. (30) ‡	High (4)	—	—	—	—	M	—
Lee and Ross (50)	Moderate (3)	—	—	—	—	M	—
McEwen et al. (44)	Low (0)	—	—	S	—	—	—
O’Keeffe et al. (48)	High (5)	—	—	—	—	S	—
Park et al. (46)	Moderate (3)	—	—	—	S	S	—
Park et al. (28)†	Moderate (3)	M	—	—	—	—	—
Park et al. (29)†	High (5)	M	—	—	—	—	—
Paternotte et al. (27)	Low (1)	—	—	—	—	—	C
Perry et al. (43)	Low (0)	—	—	M	—	—	—
Rich et al. (40)	High (5)	—	C	—	—	—	—
Rich et al. (45)	High (5)	—	—	—	M	—	—
Ross et al. (51)	Low (2)	—	S	—	—	—	S
Schultz and Griffiths (42)	Low (0)	—	—	M	—	—	—
Schut et al. (37)	High (5)	—	C	—	—	—	M
Woodworth et al. (18)	Moderate (3)	—	—	—	—	M	—

S, supportive; M, mixed; C, challenging; —, not addressed. † Milestone-based CBME subgroup; ‡ single-time-point assessment.

### 3.2 Competency development outcomes

Direct evidence of competency development was limited to four studies, all employing observational designs without pre-implementation baselines. Two of these evaluated purposefully designed PA systems (32, 33); the other two analyzed milestone-based trajectories within regulated competency-based training without an explicitly designed PA system (28, 29) and are reported here as a distinct subgroup, consistent with Section 2.3.

#### 3.2.1 Purposefully designed PA systems

Ashman et al. (32) evaluated the Australian Orthopedic Association competency-based training program (AOA21), drawing on training-information system data from 571 trainees and 46,180 workplace-based assessments accumulated between 2017 and 2022. Fellowship examination pass rates improved significantly following the transition to competency-based training (66 to 82%, *p* = 0.004), and annual performance interventions decreased from 29 to 14 (*p* = 0.01). The questionnaire component of the evaluation, however, achieved only an 18% response rate (322 of 1,778 invited), tempering the strength of stakeholder-experience inferences from this study. Findings were also mixed, with stakeholders reporting variability in delivery and perceiving assessment as onerous. Chan et al. (33) provided indirect evidence, reporting that the quality of end-of-rotation competency reports approximately doubled following implementation of a PA system (median Completed Clinical Evaluation Report Rating (CCERR) score 13.8 vs. 27.5 out of 45, *p* < 0.001, based on 25 randomly selected reports from a pre-implementation year and 25 from an early implementation year), indicating improved documentation of competency judgments rather than competency development per se. The source does not specify the statistical test underlying this comparison and reports no confidence interval for the median difference; values are reported here as published (the interquartile range reported for the post-implementation median, 20.5–23.5, is internally inconsistent with the median of 27.5, as published). Notably, the two raters who scored end-of-rotation reports were the program’s developers and the paper’s own authors, not blinded outside reviewers; this raises a potential confirmatory-rating bias that the source paper does not address.

#### 3.2.2 Milestone-based CBME without explicit PA design

Two studies examined milestone-based trajectories at contrasting scales, one at the national level (29) and one in a single institutional cohort (28). Park et al. (29) (national ACGME family medicine cohort, *n* = 3,872 across 514 programs), analyzing milestone-based progression, found that milestone ratings increased by a mean of 0.55 units per reporting period (*p* < 0.001), with 74% of residents reaching the expected graduation target across all subcompetencies. Park et al. (28) (single-institution internal medicine cohort, *n* = 34) observed statistically significant milestone progression across five reporting periods (*p* < 0.001). Both studies noted important limitations, including single-cohort designs without pre-implementation baselines and confounding with developmental maturation during training (cf. Edgar et al. on milestones evidence (34)). As specified in Section 2.8, a more fundamental concern applies to both. This is construct circularity, whereby the assessment process both generates the underlying ratings and defines the outcome (milestone level reached). Without independent convergent evidence — for example, from patient-care outcomes, blinded performance examinations, or comparison with time-based historical cohorts — observed progression cannot be cleanly distinguished from internal measurement consistency, rater frame-of-reference drift, and committee calibration (35, 36). This is a question about what milestone-trajectory evidence can support, not a dismissal of milestone systems as data sources.

Across the four studies, competency ratings rose over training in both designed PA systems and milestone-based training, but the absence of controlled designs precludes causal attribution to the assessment system itself.

### 3.3 Implementation and stakeholder experience

Sixteen studies examined how PA systems function in practice, providing essential context for understanding the limited direct competency development evidence identified above.

#### 3.3.1 Stakeholder engagement and assessment culture

How trainees and faculty engage with PA directly shapes its potential to support competency development. Schut et al. (37), in a constructivist grounded-theory study of 26 learners across five medical education programs in three countries (11 of the 26 participants were postgraduate trainees, drawn from two family medicine residency programs in Canada and the Netherlands; the remainder were undergraduate learners), found that learners’ sense of control over the assessment process was the central construct influencing their experience. Notably, learners perceived assessment stakes as a dichotomy — consequential or not — rather than the theoretically proposed continuum. Beyond a threshold, additional assessments were experienced as a checkbox exercise rather than a learning opportunity. Because the published findings are not stratified by training stage, they may partly reflect undergraduate contexts, and they are interpreted here accordingly. Gauthier et al. (38) identified a similar tension. Residents initiated workplace-based assessments primarily to meet promotion requirements rather than to seek feedback for learning, with avoidance behaviors emerging when residents were uncertain about their performance. Acai et al. (39), studying a mature system several years post-implementation, found that faculty perceived PA as having contributed to a positive culture shift in their department. Yet narrative comments remained underutilized, and both residents and faculty engaged in gaming behaviors such as selecting lenient assessors or easier clinical tasks. Faculty reluctance to document constructive feedback was reported in two included studies: Rich et al. (40) found that frontline faculty were comfortable with direct observation and oral coaching but feared that written negative feedback could jeopardize their own reappointment, leading to reliance on informal hallway conversations that bypassed the formal system; and Ashman et al. (32) similarly reported that some assessors failed to fail underperforming trainees and that the assessment process was perceived as onerous. This pattern was independently documented by Caretta-Weyer et al. (41) (Moderate quality, Met/5 = 3) across eight emergency medicine programs, where faculty reticence to provide written (as opposed to verbal) feedback was identified as a recurring implementation barrier; the convergence across three independently designed studies in different specialties and countries suggests this is a systemic feature of PA implementation rather than an idiosyncratic local finding.

#### 3.3.2 Implementation feasibility and resource requirements

The resource investment required for PA was substantial. Schultz et al. (42) (Low quality, Met/5 = 0, an innovation report with descriptive implementation data) documented the costs at one Canadian family medicine program: one day per week of program director time, 0.5 days per week each for curriculum and assessment directors, 36 h per year per academic advisor, and approximately 600 h of initial software development with 150–250 h of annual maintenance. Caretta-Weyer et al. (41) reported program leadership time of 4–30 h per month across eight emergency medicine programs (the study’s abstract reports 4–21.4 h per month; the fuller range from its results section is used here), with resident participation rates of 50–100% and faculty participation of 22–100%. Perry et al. (43) (Low quality, Met/5 = 0) described a multi-source Master Assessment Plan mapping 22 assessment types to the patient care competency alone (with additional assessments mapped to other competencies), with faculty development and stakeholder buy-in identified as ongoing challenges. Despite these demands, programs that invested in sustained implementation reported positive indicators: McEwen et al. (44) (Low quality, Met/5 = 0) documented field note completion rising from approximately 8 per resident annually before electronic implementation to 59 four years later, with over 23,000 field notes accumulated across the program.

#### 3.3.3 Competency decision-making and problem identification

Rich et al. (45), studying four Canadian programs across different specialties, identified four resident archetypes that differentially influenced competence committee functioning. High-performing engaged residents received minimal individualized feedback beyond confirmation of progress, while weakly performing residents consumed disproportionate committee time and prompted reliance on less defensible informal data sources. Park et al. (46) (single-institution internal medicine cohort, n = 142, end-of-rotation evaluation validity; from the same internal medicine program as the longitudinal cohort in (28), with which it partially overlaps), in a study of 2,701 end-of-rotation evaluations, demonstrated that PA scores could predict problem-resident identification (OR = 5.82, 95% CI 4.19–8.09, *p* < 0.001), with overall aggregated reliability of *Φ* = 0.71; the authors recommended at least 15 ratings per learner to exceed Φ = 0.70 (and ≥25 ratings to exceed Φ = 0.80).

#### 3.3.4 Measurement properties supporting competency decisions

Several studies examined whether assessment instruments within programmatic systems produce data of sufficient quality to inform competency judgments; validity in workplace-based assessment is itself an expanding conceptual field (47). O’Keeffe et al. (48) reported a composite reliability of 0.89 across workplace-based assessments, objective structured clinical examinations (OSCEs), and interviews in an Irish surgical training program, providing evidence that multi-method assessment can yield dependable scores. Woodworth et al. (18), piloting a revised assessment system across seven anesthesiology programs, achieved 99% coverage of level 1–4 milestones and found that 21 of 25 EPAs showed significant increases in entrustment-supervision ratings across training years, consistent with the developmental-trajectory pattern expected if the rating scales are tracking growing learner readiness (49). Agreement between system-generated scores and competence committee ratings, however, was assessed in a separate, single-site analysis with a different denominator. Of 23 subcompetencies examined at one program (*n* = 13, 12, and 13 residents in the three clinical anesthesia years, respectively), 15 showed a significant overall correlation between system and committee scores, 8 had correlation coefficients below 0.30 (poor correlation under the study’s interpretive bands), and only one (the patient-care subcompetency PC-8; intraclass correlation coefficient (ICC) = 0.63) reached the study’s good-reliability band (ICC 0.61–0.75). The 25 EPAs and the 23 subcompetencies are thus different analytic units from different samples (a multi-site pilot versus a single-site committee-comparison subsample), and the reliability estimates were further constrained by the late deployment of nontechnical skills assessments (NTSAs) and OSCEs only in the final 4 months of the 24-month pilot.

Two studies documented notable discordance between assessment sources or modalities with direct implications for the validity of competency inferences. Lee et al. (50) reported a near-zero association (Cramér’s V = 0.055, *p* < 0.001) between fieldnote source (resident self-assessment vs. preceptor assessment) and the progress level selected, indicating that self- and preceptor-generated ratings showed only weak association at the population level, despite the source paper’s overall framing of “reasonable consistency” between the two rating sources; second-year residents in particular rated themselves at the highest progress level significantly more often (60.6%) than first-year residents (44.5%). Self-assessment, on this evidence, cannot be treated as an interchangeable source of competency evidence within a programmatic system; it functions as a distinct reflective input that must be triangulated against external observations. Hauff et al. (30) demonstrated that assessment modality choice substantively shaped what competency gaps were detected. A multi-station standardized performance-based assessment showed intern competency rates ranging from 48 to 93% across Level 1 milestones, with notable shortfalls in aseptic technique and hand-off communication, whereas global monthly workplace evaluations rated essentially all interns as having met Level 1 milestones. The contrast between performance-based detection of domain-specific competency shortfalls and the near-uniform “pass” produced by global workplace ratings suggests that the two modalities assess different dimensions of performance, with direct implications for assessment validity and triangulation design. Reliance on a single modality within a programmatic system risks either systematically overlooking competency shortfalls or misattributing contextual performance to stable ability. Put plainly, accumulating data points does not by itself make the resulting decisions defensible; what matters is which complementary modalities are combined and how they are triangulated.

#### 3.3.5 Cross-system and contextual variation

Ross et al. (51) (Low quality, Met/5 = 2) meta-evaluated the Canadian Continuous Reflective Assessment for Training (CRAFT) framework, implemented across all 17 Canadian family medicine residency programs, drawing on documentary, survey, and program-level data. More than 85% of graduating residents in each annual cohort (2015–2019) agreed that they had had many informal opportunities for feedback during training, suggesting the formative-feedback orientation of PA had been broadly realized at the system level. However, Paternotte et al. (27) (Low quality, Met/5 = 1, an auto-ethnographic account) highlighted that core constructs such as entrustment were interpreted fundamentally differently across countries, reflecting genuine independence in the Netherlands versus expected progression under continued supervision in Canada. Cultural, legal, and licensing contexts evidently shape how PA functions regardless of shared theoretical foundations.

## 4 Discussion

This review synthesized empirical evidence on PA in PGME and yielded three principal observations. First, direct evidence of competency development was scarce. Only 4 of 20 studies measured competency-related outcomes, all observational single-cohort designs without pre-implementation comparators, and only two evaluated purposefully designed PA systems; this constrains any conclusion about effectiveness. Second, the evidence base was dominated by studies of implementation processes, stakeholder perceptions, and measurement properties, indicating that empirical inquiry has concentrated on understanding system functioning rather than testing educational impact. Third, the same implementation problems recurred across settings and specialties: barriers to feedback documentation, misalignment between intended and perceived assessment stakes, variable stakeholder engagement, and substantial resource requirements. Their consistency suggests these are inherent features of PA implementation rather than context-specific difficulties.

Beyond these implementation challenges, the outcome evidence itself is concentrated at the lower educational-outcome levels. Mapped to the New World Kirkpatrick Model (22), most studies reported stakeholder reactions (Level 1) or learning outcomes through milestone progression and assessment scores (Level 2); only one approached behavioral change (32), and none examined patient-care outcomes (Level 4). The included studies concern implementation more than impact (in Proctor et al.’s terms (24), chiefly acceptability, feasibility, and fidelity), so the field knows more about whether PA can be implemented than about what it achieves; Moore et al.’s outcomes framework (25) may map PA outcomes more directly in future work. This echoes Schumacher et al. (36), who note that links between medical education, quality of care, and patient outcomes have advanced minimally over four decades, and the Ottawa 2020 caLL to evaluate PA’s impact on learner competency and healthcare outcomes (13, 14) remains largely unanswered.

The Ottawa consensus also cautioned that partial implementation of PA may produce unwanted side effects (13), a concern surfaced here in three forms: learners conceptualized stakes as a dichotomy rather than a continuum, perceiving low-stakes assessments as high-stakes (37); assessment-seeking was driven by promotion requirements rather than learning goals, limiting learner agency (38); and faculty avoided documenting constructive feedback within the formal system due to professional consequences, undermining the meaningful feedback the model depends on (40). The conditions under which PA is theorized to support competency development — a continuum of stakes, learner agency, and meaningful feedback — are thus not yet consistently realized in the settings studied.

These postgraduate findings closely mirror the undergraduate experience documented by Heeneman et al. (15), the closest empirical comparator to this review, which found that the theory of PA and its practice diverged substantially in an undergraduate medical program. Assessments intended as low-stakes were experienced as high-stakes, and feedback use depended heavily on perceived consequences. The convergence between that undergraduate evidence and the three postgraduate patterns described above suggests that the gap between PA’s theoretical design and its lived enactment is systemic across the training continuum rather than an artifact of either training stage. This cross-stage consistency strengthens the case that implementation fidelity, not theoretical inadequacy, is the field’s central challenge.

These findings have implications for practice and policy. The persistent gap between intended and perceived stakes suggests that simply designating assessments as low-stakes is insufficient; programs may need deliberate stakeholder orientation. Faculty development was identified as a critical enabler in two included studies (32, 40) and warrants further empirical investigation as a moderator of effectiveness; reluctance to document constructive feedback represents a direct threat to the data quality on which competency decisions depend. Programs should anticipate substantial sustained investment in infrastructure, personnel, and technology, and accreditation bodies should consider whether current mandates are paired with adequate guidance, resources, and realistic effectiveness expectations, since mandated requirements in PGME do not automatically yield their intended outcomes (52).

Equity deserves separate treatment. Two concerns surface directly in the included studies: weakly performing residents absorbed disproportionate competence committee attention and prompted reliance on less defensible informal data sources (45), raising the question of whether all learners benefit equally from the system, and faculty avoidance of documented constructive feedback (40) means some learners may never receive the written, actionable feedback the model promises. A third concern was not examined by any included study but is salient for a model that elevates narrative data and committee judgment. Such judgments may import or amplify implicit bias. Disparities in assessment and recognition by race, gender, and international medical graduate status are documented elsewhere in medical education — including disparities in honor society membership (53) and the amplification of small assessment differences into consequential outcome gaps (54) — and Lucey et al. (55) characterize achieving equity in assessment as one of the field’s “wicked problems.” Whether PA mitigates or magnifies these patterns is an empirical question no included study addressed; Schumacher et al. (36) propose that the next era of assessment be defined by ensuring high-quality, equitable patient care, and examining bias in PA’s narrative and committee-based components should be part of that agenda.

The evidence base is also geographically narrow. Most included studies (80%) came from Canada and the United States, and this concentration shapes what the field can currently know. PA was theorized at Maastricht University in the Netherlands, yet the only included study offering a Dutch perspective is a Low-quality auto-ethnography (27) — the theory’s home system is empirically near-silent in the postgraduate literature captured by this review. The United Kingdom and continental Europe are absent, Australia contributes a single study (32), and Latin America, Asia, and Africa are entirely unrepresented. This matters because the included North American studies predominantly evaluate accreditation-driven, EPA- and milestone-structured implementations (Competence by Design in Canada; the ACGME milestone system in the United States), whereas the European tradition that produced PA emphasizes portfolio-based, mentor-supported designs; Paternotte et al. (27) show that even the core construct of entrustment is interpreted differently across these contexts. Evidence generated in one regulatory and cultural configuration therefore cannot be assumed to transfer to another, and the field lacks empirical insight into how PA functions in lower-resource settings, where its substantial documented resource requirements may be prohibitive.

All four competency-outcome studies relied on system-internal metrics such as milestone ratings and examination pass rates, with no included study measuring clinical performance or patient care outcomes — a gap Torre et al. (14) also identify as an explicit research priority. PA was operationalized differently across studies, shaped by educational setting, regulatory framework, and accreditation context, meaning implementation outcomes, stakeholder satisfaction, and competency findings from one setting cannot be assumed to transfer to another. Emerging technological solutions are relevant to the implementation barriers documented here. Mobile assessment platforms (16) may lower the per-encounter burden of capturing workplace-based assessments, and natural-language-processing tools that organize narrative feedback for competence committees (17) target precisely the documentation and synthesis bottlenecks that three included studies identified as dominant faculty barriers. Both were excluded from this review because they evaluate enabling tools rather than full assessment systems, and their effects on system-level outcomes remain untested; a focused review of digital enablers of PA, and primary studies testing whether such tools change documentation behavior and decision quality, would complement the system-level evidence synthesized here. Future research should also evaluate whether adherence to established implementation frameworks (e.g., Rich et al. (56)) translates into improved competency outcomes, using the implementation-outcomes vocabulary of Proctor et al. (24).

Several limitations constrain these conclusions. No included study employed a controlled design, and the four competency-outcome studies relied on single-cohort observational approaches without pre-implementation baselines, making it difficult to separate competency gains from developmental maturation. In the milestone-based studies (28, 29), construct circularity (the assessment process both generating the data and defining the outcome) further constrains causal inference. The evidence base was geographically concentrated and unevenly distributed across specialties (emergency and family medicine comprising over half of included studies), with uneven methodological quality (nine High, six Moderate, five Low). The operational mapping of studies to the four PA features (Supplementary Material 5), and the resulting subgroup designation, required judgment based on what source papers reported and may understate the design intent of under-described systems. One multinational qualitative study included both undergraduate and postgraduate participants without stratified reporting (37), so its contribution cannot be attributed exclusively to PGME. The boundary between PA and its component tools remains debated; studies evaluating single instruments in isolation were excluded unless embedded within an explicitly described programmatic system, a conceptually necessary decision that may nonetheless have omitted relevant evidence. No formal assessment of reporting or publication bias was possible given the heterogeneous, predominantly non-comparative designs; the restriction to English-language, peer-reviewed publications and the exclusion of gray literature (dissertations, governmental and institutional evaluation reports) may have systematically excluded evidence from implementation-rich settings in continental Europe, Latin America, Asia, and Africa. Finally, heterogeneity precluded meta-analysis, and confidence in the principal qualitative findings, while formally assessed, was no higher than moderate (Supplementary Material 6).

In sum, the empirical evidence for PA in PGME has not kept pace with its theoretical development or its adoption. Available research has concentrated on how these systems are implemented and experienced rather than whether they improve competency development; where competency outcomes were measured, no study provided controlled evidence attributing observed gains to the assessment system itself. Building an evidence base that matches the scale and specificity of PA’s implementation remains an important priority for the field.

## Data Availability

The original contributions presented in the study are included in the article/Supplementary material, further inquiries can be directed to the corresponding author.
