# Supplementary material for: Programmatic assessment and competency development in postgraduate medical education: a systematic review and narrative synthesis
Source: Front Med (Lausanne). 2026 Jul 16;13:1873126. doi: 10.3389/fmed.2026.1873126 (PMC13422548; doi:10.3389/fmed.2026.1873126)
Supplement: Supplementary file 1 [file Table_1.DOCX]

**Supplementary Material 1. Search strategies**

**1. MEDLINE(R) ALL (PubMed)**

Interface: PubMed (NCBI).

Date of search: December 2025. Limits: English; 2005–2025.

(("programmatic assessment"[Title/Abstract]

OR "systems of assessment"[Title/Abstract]

OR "assessment system"[Title/Abstract])

AND ("postgraduate medical education"[MeSH]

OR "graduate medical education"[Title/Abstract]

OR "residency training"[Title/Abstract])

AND ("competence"

OR "clinical performance"

OR "learning outcomes"

OR "feedback quality"))

AND ("2005"[Date - Publication] : "2025"[Date - Publication])

Records retrieved: 144. Search string as specified in the review protocol.

**2. Web of Science**

Interface: Web of Science Core Collection (Clarivate).

Date of search: December 2025. Limits: English; 2005–2025.

TS=("programmatic assessment"

OR "systems of assessment"

OR "assessment system*"

OR "assessment NEAR/3 system*"

OR "programmatic feedback"

OR "assessment program*")

AND TS=("postgraduate medical education"

OR "graduate medical education"

OR "residency training"

OR "medical residency"

OR "specialist training")

AND PY=2005-2025

AND LA=(English)

Records retrieved: 127. Search string as specified in the review protocol.

**3. Embase**

Interface: Ovid Embase.

Date of search: December 2025. Limits: English; 2005–2025.

1 ("programmatic assessment" OR "systems of assessment"

OR "assessment system" OR "assessment program*"

OR "programmatic feedback").ab,ti.

2 exp medical education/

3 ("postgraduate medical education" OR "graduate medical education"

OR "residency training" OR "medical residency"

OR "specialist training").ab,ti.

4 2 OR 3

5 (competence OR "clinical performance"

OR "learning outcomes" OR "feedback quality").ab,ti.

6 1 AND 4 AND 5

7 limit 6 to (english language and yr="2005 - 2025")

Records retrieved: 98. Adapted translation of the protocol search.¹

**4. Scopus**

Interface: Scopus (Elsevier).

Date of search: December 2025. Limits: English; 2005–2025.

TITLE-ABS-KEY(

( "programmatic assessment" OR "systems of assessment"

OR "assessment system*" OR "assessment program*"

OR "programmatic feedback" )

AND

( "postgraduate medical education" OR "graduate medical education"

OR "residency training" OR "medical residency"

OR "specialist training" )

AND

( competence OR "clinical performance"

OR "learning outcomes" OR "feedback quality" )

)

AND PUBYEAR > 2004 AND PUBYEAR < 2026

AND ( LIMIT-TO ( LANGUAGE, "English" ) )

Records retrieved: 66. Adapted translation of the protocol search.¹

**5. EBSCOhost**

Interface: EBSCOhost (Academic Search Ultimate; CINAHL Complete; ERIC; LISTA).

Date of search: December 2025. Limits: English; 2005–2025.

S1 AB ( "programmatic assessment" OR "systems of assessment"

OR "assessment system*" OR "assessment program*"

OR "programmatic feedback" )

S2 AB ( "postgraduate medical education" OR "graduate medical education"

OR "residency training" OR "medical residency"

OR "specialist training" )

S3 AB ( competence OR "clinical performance"

OR "learning outcomes" OR "feedback quality" )

S4 S1 AND S2 AND S3

Limiters: Published Date 20050101-20251231; Language = English

Records retrieved: 64 (combined across the four EBSCOhost databases). Adapted translation of the protocol search.¹

Total records identified: 499. After removal of 242 duplicates, 257 unique records advanced to title/abstract screening.

*¹ For Embase, Scopus, and EBSCOhost, the protocol search was adapted to each database’s native syntax. The strings shown are equivalent translations of the executed searches; the hit counts are the counts returned by the executed searches.*
