# Supplementary material for: Programmatic assessment and competency development in postgraduate medical education: a systematic review and narrative synthesis
Source: Front Med (Lausanne). 2026 Jul 16;13:1873126. doi: 10.3389/fmed.2026.1873126 (PMC13422548; doi:10.3389/fmed.2026.1873126)
Supplement: Supplementary file 2 [file Table_2.DOCX]

**Supplementary Material 2. Excluded full-text studies with reasons for exclusion**

A total of 29 reports assessed as full text were excluded. Reasons are summarised in Table S2.1 and listed in full in Table S2.2. Where more than one reason applied, exclusion categories were applied hierarchically and the primary reason is reported.

**Table S2.1. Summary of exclusion reasons**

| **Exclusion category** | **Studies** | **n** | **%** |
| --- | --- | --- | --- |
| Ineligible study design | Clarke, 2025; Gates, 2024; Schumacher, 2024; Booth, 2024; Warm, 2022; Cooney, 2021; McEllistrem, 2023; Rich, 2022; Woodworth, 2021; Sherbino, 2020; Pearce, 2019; Timmerman, 2017; Li, 2023; Marty, 2022 | 14 | 48.3% |
| Ineligible intervention | Ahn, 2023; Koduri, 2020; Warm, 2014; Brittlebank, 2013; Tabuenca, 2007; Savard, 2022; Martin, 2020 | 7 | 24.1% |
| Ineligible outcome | Ginsburg, 2021; Cheung, 2022; Dubois, 2021 | 3 | 10.3% |
| Ineligible publication type | Misra, 2021; Pan, 2021; Tanaka, 2021 | 3 | 10.3% |
| Ineligible population | Barbagallo, 2024 | 1 | 3.4% |
| No empirical data | Cheung, 2024 | 1 | 3.4% |
| **Total** |  | **29** | **100.0%** |

**Table S2.2. Full list of excluded studies with reasons**

| **#** | **First author, year** | **Title** | **Exclusion category** | **Brief rationale** |
| --- | --- | --- | --- | --- |
| 1 | Clarke, 2025 | The Landscape of Mandated Assessments of Surgical Skills: A Document Analysis | Ineligible study design | Document analysis of ACGME-mandated surgical skills assessment requirements across nine specialties; catalogues existing mandated assessments rather than empirically evaluating a programmatic assessment system. |
| 2 | Gates, 2024 | An Ideal System of Assessment to Support Competency-Based Graduate Medical Education: Key Attributes and Proposed Next Steps | Ineligible study design | Framework/position paper describing ideal characteristics of assessment systems and proposing next steps; conceptual rather than empirical. |
| 3 | Barbagallo, 2024 | Implementation of Programmatic Assessment in Radiation Oncology Medical Physics Training | Ineligible population | Studies programmatic assessment in radiation oncology medical physics training; participants are medical physics trainees, not medical residents or fellows. |
| 4 | Schumacher, 2024 | The Next Era of Assessment | Ineligible study design | Conceptual/framework paper in Academic Medicine supplement discussing theoretical approaches to assessment; proposes new paradigms rather than evaluating an implemented PA system. |
| 5 | Ahn, 2023 | Only as Strong as the Weakest Link: Cross-Sectional Study of Residents’ Ratings of the Assessment System | Ineligible intervention | Evaluates resident perceptions of their general assessment system, not a comprehensive programmatic assessment system with multiple integrated tools, competence committees, and longitudinal tracking. |
| 6 | Booth, 2024 | Fine-Tuning Large Language Models to Enhance Programmatic Assessment in Graduate Medical Education | Ineligible study design | Tool development/technical implementation paper focused on optimizing NLP algorithms for organizing narrative feedback; lacks empirical evaluation of the PA system’s impact on learner outcomes. |
| 7 | Warm, 2022 | The Education Passport: Connecting Programmatic Assessment Across Learning and Practice | Ineligible study design | Describes the conceptual framework and implementation of the Education Passport concept; focuses on design and explanation without presenting empirical evaluation data. |
| 8 | Cooney, 2021 | The Emergency Medicine Milestones 2.0 | Ineligible study design | Framework paper describing the updated EM Milestones 2.0 competency framework; does not present empirical research evaluating a programmatic assessment system. |
| 9 | McEllistrem, 2023 | Exploring the Irish GP Training Programme’s Assessment System | Ineligible study design | Exploratory perception study of an assessment system without evaluating implementation outcomes or competency development. |
| 10 | Rich, 2022 | Operationalizing Programmatic Assessment: The CBME Programmatic Assessment Practice Guidelines | Ineligible study design | Guideline development paper using focus groups, stakeholder consultations, and expert review to create PA practice guidelines; does not present empirical evaluation of outcomes or impact on trainees. |
| 11 | Ginsburg, 2021 | Numbers Encapsulate, Words Elaborate: Toward the Best Use of Comments for Assessment and Feedback on Entrustment Ratings | Ineligible outcome | Examines the complementary roles of numerical ratings and narrative comments on entrustment forms; focuses on optimizing assessment tool format rather than system-level PA outcomes. |
| 12 | Misra, 2021 | The Importance of Competency-Based Programmatic Assessment in GME | Ineligible publication type | Editorial/perspectives piece discussing the importance of competency-based PA; does not present original empirical research. |
| 13 | Woodworth, 2021 | Development and Pilot Testing of Entrustable Professional Activities for US Anesthesiology Residency Training | Ineligible study design | Tool development and pilot testing paper using Delphi methodology to create EPAs; develops assessment tools but lacks evaluation of a comprehensive PA system’s impact. |
| 14 | Koduri, 2020 | Using a Mobile Application for Evaluation of Procedural Learning in Neurosurgery | Ineligible intervention | Evaluates the SIMPL mobile application for case-specific surgical competency assessments; studies a single assessment tool used in isolation, not a comprehensive, longitudinal PA system. |
| 15 | Sherbino, 2020 | The Competency-Based Medical Education Evolution of Canadian Emergency Medicine Specialist Training | Ineligible study design | Descriptive paper outlining the rationale, process, and design features of a national CBME model; describes the framework without presenting empirical evaluation data. |
| 16 | Pearce, 2019 | When I Say… Programmatic Assessment in Postgraduate Medical Education | Ineligible study design | ‘When I say…’ series article providing a narrative definition and explanation of PA; educational/definitional commentary rather than empirical research. |
| 17 | Timmerman, 2017 | A Practical Approach to Programmatic Assessment Design | Ineligible study design | Case study analysis presenting four guiding steps for PA redesign in a Dutch residency; primarily a descriptive framework and practical guidance paper rather than empirical research evaluating PA outcomes. |
| 18 | Warm, 2014 | Entrustment and Mapping of Observable Practice Activities for Resident Assessment | Ineligible intervention | Describes a system using observable practice activities mapped to milestones and EPAs; focuses on assessment tool development and methodology rather than evaluation of a comprehensive PA system. |
| 19 | Brittlebank, 2013 | Workplace-Based Assessments in Psychiatry: Evaluation of a Whole Assessment System | Ineligible intervention | Evaluates individual WBA tools (CBD, Mini-CEX, Mini-PAT, DOPS) as separate instruments in psychiatric training; assesses feasibility/popularity of individual tools rather than an integrated PA system with developmental tracking. |
| 20 | Tabuenca, 2007 | Multi-Institutional Validation of a Web-Based Core Competency Assessment System | Ineligible intervention | Validates a web-based system for end-of-rotation faculty ratings of ACGME core competencies; focuses on a single assessment tool rather than a programmatic system integrating multiple assessment methods. |
| 21 | Li, 2023 | Perfecting Summative Assessment System for Standardized GP Residency Training in China | Ineligible study design | Uses Delphi method with 32 expert panelists to develop summative assessment indicators; expert consensus technique, not empirical research evaluating a PA system’s implementation or outcomes. |
| 22 | Cheung, 2022 | Ready, Set, Go! Evaluating Readiness to Implement CBME | Ineligible outcome | Evaluates organizational readiness for implementing CBME using the R=MC² framework; measures readiness perception rather than competency development or effectiveness of the PA system itself. |
| 23 | Marty, 2022 | A Mobile Application to Facilitate Implementation of Programmatic Assessment in Anaesthesia | Ineligible study design | Technology development paper describing the creation of a mobile application for PA; focuses on tool development rather than empirical evaluation of PA system outcomes. |
| 24 | Pan, 2021 | Opportunity Within a Crisis — A Push Towards Programmatic Assessment in COVID-19 | Ineligible publication type | Narrative commentary describing a PA implementation during COVID-19; presents reflections on experience without systematic empirical research design. |
| 25 | Savard, 2022 | Development and Use of a Computerized System to Track Competency Development of FM Residents | Ineligible intervention | Develops and evaluates a single computerized tracking system for competency development; focuses on one technology tool’s convergence with assessor decisions, not a comprehensive multi-tool PA system. |
| 26 | Tanaka, 2021 | Entrustable Professional Activities as a Framework for Workplace-Based Assessment in GME | Ineligible publication type | Doctoral dissertation (PhD thesis) rather than a peer-reviewed journal article; does not meet publication type inclusion criterion. |
| 27 | Dubois, 2021 | Validity of Entrustment Scales Within Anesthesiology Residency Training | Ineligible outcome | Examines psychometric properties of entrustment rating scales as a single assessment tool; focuses on validity evidence for one instrument rather than system-level PA outcomes. |
| 28 | Martin, 2020 | The Impact of Entrustment Assessments on Feedback and Learning: Trainee Perspectives | Ineligible intervention | Studies trainee perspectives on entrustment assessments as a single assessment modality; does not evaluate a comprehensive, integrated programmatic assessment system. |
| 29 | Cheung, 2024 | Design and Implementation of a National Program of Assessment Model — Integrating Entrustable Professional Activity Assessments in Canadian Specialist Postgraduate Medical Education | No empirical data | Initially advanced to data extraction (Study ID S05); on closer reading the paper was identified as a descriptive/reflective account of a national program of assessment model without empirical outcome data on learner competency development, and was therefore excluded from the synthesis. |
