# Supplementary material for: Programmatic assessment and competency development in postgraduate medical education: a systematic review and narrative synthesis
Source: Front Med (Lausanne). 2026 Jul 16;13:1873126. doi: 10.3389/fmed.2026.1873126 (PMC13422548; doi:10.3389/fmed.2026.1873126)
Supplement: Supplementary file 3 [file Table_3.DOCX]

# Supplementary Material 3. PRISMA 2020 Checklist

Programmatic Assessment and Competency Development in Postgraduate Medical Education: A Systematic Review and Narrative Synthesis (PROSPERO CRD420261349850).

This checklist follows Page MJ, McKenzie JE, Bossuyt PM, et al. The PRISMA 2020 statement: an updated guideline for reporting systematic reviews. BMJ 2021;372:n71. doi:10.1136/bmj.n71. For each item, the column “Location reported” indicates the section, paragraph, table, figure, or supplementary material of this manuscript in which the corresponding item is addressed.

| **#** | **Section and Topic** | **Checklist item** | **Location reported** |
| --- | --- | --- | --- |
| **TITLE** | | | |
| 1 | Title | Identify the report as a systematic review. | Title page |
| **ABSTRACT** | | | |
| 2 | Abstract | See the PRISMA 2020 for Abstracts checklist. | Abstract |
| **INTRODUCTION** | | | |
| 3 | Rationale | Describe the rationale for the review in the context of existing knowledge. | Introduction |
| 4 | Objectives | Provide an explicit statement of the objective(s) or question(s) the review addresses. | Introduction (final paragraph) |
| **METHODS** | | | |
| 5 | Eligibility criteria | Specify the inclusion and exclusion criteria for the review and how studies were grouped for the syntheses. | Methods Section 2.2, Section 2.3 |
| 6 | Information sources | Specify all databases, registers, websites, organisations, reference lists and other sources searched or consulted to identify studies. Specify the date when each source was last searched or consulted. | Methods Section 2.4; Supplementary Material 1 |
| 7 | Search strategy | Present the full search strategies for all databases, registers and websites, including any filters and limits used. | Methods Section 2.4; Supplementary Material 1 |
| 8 | Selection process | Specify the methods used to decide whether a study met the inclusion criteria of the review, including how many reviewers screened each record and each report retrieved, whether they worked independently, and, if applicable, details of automation tools used in the process. | Methods Section 2.5 |
| 9 | Data collection process | Specify the methods used to collect data from reports, including how many reviewers collected data from each report, whether they worked independently, any processes for obtaining or confirming data from study investigators, and, if applicable, details of automation tools used in the process. | Methods Section 2.6 |
| 10a | Data items | List and define all outcomes for which data were sought. Specify whether all results that were compatible with each outcome domain in each study were sought, and if not, the methods used to decide which results to collect. | Methods Section 2.3, Section 2.6 |
| 10b | Data items | List and define all other variables for which data were sought (e.g. participant and intervention characteristics, funding sources). Describe any assumptions made about any missing or unclear information. | Methods Section 2.6; Table 1 |
| 11 | Study risk of bias assessment | Specify the methods used to assess risk of bias in the included studies, including details of the tool(s) used, how many reviewers assessed each study and whether they worked independently, and if applicable, details of automation tools used in the process. | Methods Section 2.7 |
| 12 | Effect measures | Specify for each outcome the effect measure(s) (e.g. risk ratio, mean difference) used in the synthesis or presentation of results. | Methods Section 2.8 (narrative synthesis; no pooled effect measures computed) |
| 13a | Synthesis methods | Describe the processes used to decide which studies were eligible for each synthesis (e.g. tabulating the study intervention characteristics and comparing against the planned groups for each synthesis). | Methods Section 2.8 |
| 13b | Synthesis methods | Describe any methods required to prepare the data for presentation or synthesis, such as handling of missing summary statistics, or data conversions. | Methods Section 2.8 (no data conversions required) |
| 13c | Synthesis methods | Describe any methods used to tabulate or visually display results of individual studies and syntheses. | Methods Section 2.8; Table 1; Results Section 3 |
| 13d | Synthesis methods | Describe any methods used to synthesize results and provide a rationale for the choice(s). If meta-analysis was performed, describe the model(s), method(s) to identify the presence and extent of statistical heterogeneity, and software package(s) used. | Methods Section 2.8 (narrative synthesis following Popay et al.; meta-analysis not performed due to heterogeneity) |
| 13e | Synthesis methods | Describe any methods used to explore possible causes of heterogeneity among study results (e.g. subgroup analysis, meta-regression). | Methods Section 2.8; Results Section 3 (heterogeneity explored narratively by design, setting, and outcome domain) |
| 13f | Synthesis methods | Describe any sensitivity analyses conducted to assess robustness of the synthesized results. | Not applicable (narrative synthesis) |
| 14 | Reporting bias assessment | Describe any methods used to assess risk of bias due to missing results in a synthesis (arising from reporting biases). | No formal assessment of risk of bias due to missing results (publication or selective-reporting bias) was undertaken: the heterogeneous, predominantly non-comparative designs precluded funnel-plot-based or statistical approaches. This is acknowledged as a limitation in the Discussion (Limitations paragraph). MMAT appraisal (Methods Section 2.7) addressed within-study data completeness only, which is distinct from synthesis-level reporting bias. |
| 15 | Certainty assessment | Describe any methods used to assess certainty (or confidence) in the body of evidence for an outcome. | Methods Section 2.9: confidence in the principal qualitative and implementation findings was assessed using GRADE-CERQual (evidence profile in Supplementary Material 6). GRADE was not applicable to the quantitative strand (heterogeneous descriptive designs without pooled or comparable effect estimates); certainty in quantitative findings is discussed narratively in relation to design limitations. |
| **RESULTS** | | | |
| 16a | Study selection | Describe the results of the search and selection process, from the number of records identified in the search to the number of studies included in the review, ideally using a flow diagram. | Results Section 3.1; Figure 1 (PRISMA flow diagram) |
| 16b | Study selection | Cite studies that might appear to meet the inclusion criteria, but which were excluded, and explain why they were excluded. | Supplementary Material 2 (excluded studies with reasons) |
| 17 | Study characteristics | Cite each included study and present its characteristics. | Results Section 3.1; Table 1 |
| 18 | Risk of bias in studies | Present assessments of risk of bias for each included study. | Results Section 3.1; Table 1 (MMAT quality ratings) See Supplementary Material 4 for adjudicated scorecards. |
| 19 | Results of individual studies | For all outcomes, present, for each study: (a) summary statistics for each group (where appropriate) and (b) an effect estimate and its precision (e.g. confidence/credible interval), ideally using structured tables or plots. | Results Sections 3.2–3.3; Tables 1–2 |
| 20a | Results of syntheses | For each synthesis, briefly summarize the characteristics and risk of bias among contributing studies. | Results Sections 3.2–3.3; Table 2 |
| 20b | Results of syntheses | Present results of all statistical syntheses conducted. If meta-analysis was done, present for each the summary estimate and its precision (e.g. confidence/credible interval) and measures of statistical heterogeneity. If comparing groups, describe the direction of the effect. | Not applicable (meta-analysis not performed); narrative results in Results Sections 3.2–3.3; Table 2 |
| 20c | Results of syntheses | Present results of all investigations of possible causes of heterogeneity among study results. | Results Sections 3.2–3.3 (heterogeneity by design, setting, and outcome domain discussed narratively) |
| 20d | Results of syntheses | Present results of all sensitivity analyses conducted to assess the robustness of the synthesized results. | Not applicable |
| 21 | Reporting biases | Present assessments of risk of bias due to missing results (arising from reporting biases) for each synthesis assessed. | Discussion, Limitations paragraph: the absence of a formal reporting-bias assessment is presented and discussed, including potential publication bias, the English-language restriction, and the exclusion of grey literature (dissertations and governmental/institutional evaluation reports). |
| 22 | Certainty of evidence | Present assessments of certainty (or confidence) in the body of evidence for each outcome assessed. | Methods Section 2.9; Supplementary Material 6 (GRADE-CERQual evidence profile per finding); Limitations paragraph. GRADE was not applicable to the quantitative strand (no pooled or comparable effect estimates). |
| **DISCUSSION** | | | |
| 23a | Discussion | Provide a general interpretation of the results in the context of other evidence. | Discussion paragraphs 1–4 (principal observations; outcome-level mapping; partial-implementation effects; comparison with undergraduate evidence) |
| 23b | Discussion | Discuss any limitations of the evidence included in the review. | Discussion, Limitations paragraph (penultimate paragraph) |
| 23c | Discussion | Discuss any limitations of the review processes used. | Discussion, Limitations paragraph (penultimate paragraph) |
| 23d | Discussion | Discuss implications of the results for practice, policy, and future research. | Discussion paragraphs 5–8 (practice and policy; equity; geographic transferability; future research and digital enablers) |
| **OTHER INFORMATION** | | | |
| 24a | Registration and protocol | Provide registration information for the review, including the register name and registration number, or state that the review was not registered. | Methods Section 2.1 (PROSPERO CRD420261349850) |
| 24b | Registration and protocol | Indicate where the review protocol can be accessed, or state that a protocol was not prepared. | Methods Section 2.1 (PROSPERO record) |
| 24c | Registration and protocol | Describe and explain any amendments to information provided at registration or in the protocol. | Risk-of-bias appraisal: a fresh dual-reviewer + adjudicator MMAT 2018 pass was performed in May 2026 to ensure transparency and reproducibility; this is documented in Supplementary Material 4 (per-study scorecards, disagreement log, inter-rater agreement) and is best understood as a methodological refinement rather than a substantive protocol amendment, since the appraisal tool (MMAT 2018) and the high/moderate/low cut-points were unchanged. Separately, four operational clarifications introduced during peer review (eligibility wording for systematic reviews and evaluation reports; the operational definition of programmatic assessment with the milestone-based CBME subgroup; the specification of construct circularity as a validity threat and the descriptive outcome-level mapping; and the addition of a GRADE-CERQual certainty assessment) are reported transparently in the manuscript (Section 2.1) and were not part of the originally registered protocol (PROSPERO CRD420261349850). |
| 25 | Support | Describe sources of financial or non-financial support for the review, and the role of the funders or sponsors in the review. | Funding statement (title page / acknowledgments) |
| 26 | Competing interests | Declare any competing interests of review authors. | Declarations / Competing interests statement |
| 27 | Availability of data, code and other materials | Report which of the following are publicly available and where they can be found: template data collection forms; data extracted from included studies; data used for all analyses; analytic code; any other materials used in the review. | Supplementary Materials 1–6 accompany the manuscript. The data extraction workbook, MMAT scorecards with quoted-evidence justifications, the disagreement log, the programmatic assessment fidelity matrix, and the GRADE-CERQual evidence profiles are available from the corresponding author on reasonable request. The review protocol is registered with PROSPERO (CRD420261349850). |

**Source citation**

Page MJ, McKenzie JE, Bossuyt PM, Boutron I, Hoffmann TC, Mulrow CD, Shamseer L, Tetzlaff JM, Akl EA, Brennan SE, Chou R, Glanville J, Grimshaw JM, Hróbjartsson A, Lalu MM, Li T, Loder EW, Mayo-Wilson E, McDonald S, McGuire LA, Stewart LA, Thomas J, Tricco AC, Welch VA, Whiting P, Moher D. The PRISMA 2020 statement: an updated guideline for reporting systematic reviews. BMJ. 2021;372:n71. doi:10.1136/bmj.n71.
