# Supplementary material for: Programmatic assessment and competency development in postgraduate medical education: a systematic review and narrative synthesis
Source: Front Med (Lausanne). 2026 Jul 16;13:1873126. doi: 10.3389/fmed.2026.1873126 (PMC13422548; doi:10.3389/fmed.2026.1873126)
Supplement: Supplementary file 4 [file Table_4.DOCX]

**Supplementary Material 4. Methodological Quality Appraisal: Per-study MMAT 2018 Scorecards, Disagreement Log, and Inter-Rater Agreement**

# 1. Overview

All 20 studies included in this systematic review were independently appraised for methodological quality by two reviewers (Reviewer A and Reviewer B) using the Mixed Methods Appraisal Tool (MMAT) version 2018 (Hong et al., 2018), a single instrument designed to enable critical appraisal of qualitative, quantitative, and mixed-methods studies within one review. Each reviewer independently classified the study design, then rated two screening items (S1, S2) and the five category-specific items appropriate to that design (1.1–1.5 qualitative; 3.1–3.5 quantitative non-randomised; 4.1–4.5 quantitative descriptive; 5.1–5.5 mixed methods; 2.1–2.5 RCT, not applicable in this review). All disagreements — both at the design-classification and item-rating levels — were adjudicated by a third reviewer who returned to the source PDFs, examined the evidence quoted by each reviewer, and recorded a final rating with a quoted-evidence justification. Raw and quantitative inter-rater agreement statistics (item-level raw agreement, Cohen's κ, category-level agreement, Met/5 agreement, mean absolute difference between Reviewer A's and Reviewer B's Met/5 counts) are reported in Section 6 below. Full per-study scorecards with quoted-evidence justifications follow in Section 4. Study identifiers follow the project’s screening log and run from S01 to S21 (with no S05); the 20 included studies therefore carry non-consecutive identifiers.

# 2. Headline statistics

- Studies appraised: 20
- Mean of the descriptive Met/5 index (final adjudicated): 3.20 (range 0–5; reported for transparency only — the MMAT discourages summary quality scores, see note below)
- Distribution: High (Met/5 ≥ 4) = 9; Moderate (Met/5 = 3) = 6; Low (Met/5 ≤ 2) = 5
- Total item-level disagreements adjudicated: 19 (1 category-level + 18 item-level)
- Inter-rater agreement: item-level raw 86.7% (117/135); Cohen's κ = 0.707 (substantial agreement, Landis & Koch 1977); category-level 95.0% (19/20); Met/5 agreement 85.0% (17/20); mean absolute difference between Reviewer A's and Reviewer B's Met/5 counts = 0.15

# 3. Quality distribution

| **Cut-point** | **n** | **%** | **Studies (Met/5)** |
| --- | --- | --- | --- |
| High (≥4/5) | 9 | 45% | S01 (Acai 2019, 5/5), S02 (Ashman 2025, 4/5), S06 (Gauthier 2024, 5/5), S07 (Hauff 2014, 4/5), S10 (O'Keeffe 2024, 5/5), S13 (Park 2021, 5/5), S16 (Rich 2020, 5/5), S17 (Rich 2022, 5/5), S20 (Schut 2018, 5/5) |
| Moderate (3/5) | 6 | 30% | S03 (Caretta-Weyer 2025, 3/5), S04 (Chan 2015, 3/5), S08 (Lee 2022, 3/5), S11 (Park 2016, 3/5), S12 (Park 2020, 3/5), S21 (Woodworth 2024, 3/5) |
| Low (≤2/5) | 5 | 25% | S09 (McEwen 2015, 0/5), S14 (Paternotte 2024, 1/5), S15 (Perry 2018, 0/5), S18 (Ross 2023, 2/5), S19 (Schultz 2016, 0/5) |
| MMAT Category Distribution (final adjudicated) |  |  |  |
| Category (n) | Studies |  |  |
| Category 1 — Qualitative (6) | S01 (Acai 2019), S06 (Gauthier 2024), S14 (Paternotte 2024), S16 (Rich 2020), S17 (Rich 2022), S20 (Schut 2018) |  |  |
| Category 3 — Quantitative non-randomized (2) | S04 (Chan 2015), S08 (Lee 2022) |  |  |
| Category 4 — Quantitative descriptive (9) | S03 (Caretta-Weyer 2025), S07 (Hauff 2014), S10 (O'Keeffe 2024), S11 (Park 2016), S12 (Park 2020), S13 (Park 2021), S15 (Perry 2018), S19 (Schultz 2016), S21 (Woodworth 2024) |  |  |
| Category 5 — Mixed methods (3) | S02 (Ashman 2025), S09 (McEwen 2015), S18 (Ross 2023) |  |  |

# 4. Per-study final adjudicated MMAT scorecards

For each included study, the table below reports both reviewers' independent ratings, the FINAL adjudicated rating, and the quoted-evidence justification (with page/section reference). Where Reviewer A and Reviewer B disagreed, the adjudication rationale is reproduced beneath the Final justification. Quotations have been truncated to approximately 250 characters where necessary; truncation is indicated by '…'.

## S01 — Acai 2019

**Study ID:** S01 **Citation:** Acai 2019 (Canada, Emergency Medicine) **Sample:** 16 attending EM physicians (1 program)

**MMAT category (final adjudicated):** Category 1 — Qualitative **Met/5 (final adjudicated):** 5 **Quality category:** High (5/5) **Disagreements adjudicated:** 0

| **Item** | **Item name** | **Reviewer A** | **Reviewer B** | **Final** | **Final justification (quoted evidence; adjudication rationale where ratings differed)** |
| --- | --- | --- | --- | --- | --- |
| S1 | Are there clear research questions? | Y | Y | Y | "we used McMAP as a frame of reference for better understanding the experiences of attending emergency physicians using programmatic WBA." [p.435 (Introduction final paragraph)] |
| S2 | Do the collected data allow to address the research questions? | Y | Y | Y | "We conducted a qualitative study that used semi-structured interviews analyzed using interpretive description to explore attending physicians' experiences using McMAP." [p.435 (Methods - Study design)] |
| 1.1 | Is the qualitative approach appropriate to answer the research question? | Y | Y | Y | "Interpretive description is a method of qualitative analysis that allows for 'the smaller scale qualitative investigation of a clinical phenomenon of interest … for the purpose of capturing themes and patterns within subjective perceptions.'" [p.435 (Methods)] |
| 1.2 | Are the qualitative data collection methods adequate to address the research question? | Y | Y | Y | "We contacted 24 potential participants via e-mail. Sampling occurred in roughly three rounds, with periods of data analysis taking place between each round. This iterative approach allowed us to gradually refine our interview questions and hone in…" [p.436 (Methods - Participant recruitment and data collection)] |
| 1.3 | Are the findings adequately derived from the data? | Y | Y | Y | "We employed interpretive description as outlined by Thorne, reading the transcripts from the first round of interviews in detail and then inductively generating codes to encompass the main ideas within each. We met regularly to compare codes and…" [p.436 (Methods - Data management and analysis)] |
| 1.4 | Is the interpretation of results sufficiently substantiated by data? | Y | Y | Y | "[McMAP provides assessors] with the framework … to look at the milestones or more pertinent things that the learner needs to be able to meet (P03)" [p.437 (Table 2 and Results)] |
| 1.5 | Is there coherence between qualitative data sources, collection, analysis and interpretation? | Y | Y | Y | "We followed a number of standard recommendations to ensure the trustworthiness of our qualitative data… we documented all stages of the analytic process and triangulated data both between researchers and across data sources (i.e., the resident…" [p.436 (Methods - Data management and analysis)] |

## S02 — Ashman 2025

**Study ID:** S02 **Citation:** Ashman 2025 (Australia, Orthopedic Surgery) **Sample:** 46,180 WBAs from 571 trainees + 322 survey responses (18%) + 9 focus groups (national)

**MMAT category (final adjudicated):** Category 5 — Mixed methods **Met/5 (final adjudicated):** 4 **Quality category:** High (4/5) **Disagreements adjudicated:** 1

| **Item** | **Item name** | **Reviewer A** | **Reviewer B** | **Final** | **Final justification (quoted evidence; adjudication rationale where ratings differed)** |
| --- | --- | --- | --- | --- | --- |
| S1 | Are there clear research questions? | Y | Y | Y | "The aims of the program evaluation were to identify whether the goal of AOA21 has been achieved, recommend interventions or changes to close any gaps in implementation of the original goal, determine if the curriculum, training structure, learning…" [p.3 (Aims)] |
| S2 | Do the collected data allow to address the research questions? | Y | Y | Y | "Materials for the review consisted of data obtained from the TIMS, in-training surveys of all trainees, AOA membership-wide online surveys, and focus group discussions. TIMS data included cumulative entries for WBAs, feedback entries and logbooks…" [p.3 (Methods)] |
| 5.1 | Is there an adequate rationale for using a mixed methods design to address the research question? | CT | Y | Y | "The review team used a mixed methods research approach, with quantitative statistical analysis of the TIMS and surveys data, which helped inform focus group questions. De-identified narrative data from the focus group transcripts and free-text…" [p.3-4 (Methods)] Adjudication: Reviewer A: Can't tell (rationale not made explicit beyond design statement); Reviewer B: Yes (sequential explanatory rationale stated). Final adjudicated rating: Yes. The article explicitly identifies the design (sequential explanatory: quantitative TIMS/survey results informed focus-group questions) and links it to the Core Components Framework (Van Melle et al. 2019). MMAT 5.1 asks for an adequate rationale for mixed methods, and the stated sequential explanatory logic satisfies it. |
| 5.2 | Are the different components of the study effectively integrated to answer the research question? | Y | Y | Y | "Survey responses from trainees, recent graduates and training faculty revealed alignment in most areas but divergent opinions in some. Detailed discussion of these discrepancies with the focus groups enabled better understanding of the impact of…" [p.1 (Results in Abstract); p.3 (Methods)] |
| 5.3 | Are the outputs of the integration of qualitative and quantitative components adequately interpreted? | Y | Y | Y | "Although the program evaluation found evidence for assessment of progression of competence and reduction in remediation of underperforming trainees with the introduction of WBAs and feedback entries, the general impression is that there are too…" [p.8 (Discussion)] |
| 5.4 | Are divergences and inconsistencies between quantitative and qualitative results adequately addressed? | Y | Y | Y | "Interestingly, although trainees do not rate WBAs and feedback entries as being highly constructive for their learning, they appreciate the importance of the regular review meetings for their development. Given that these meetings include a review…" [p.8 (Discussion)] |
| 5.5 | Do the different components of the study adhere to the quality criteria of each tradition of the methods involved? | CT | CT | CT | "Questionnaires were sent to all AOA trainees and members in 2022 (n = 1778) and 322 responses were received (a response rate of 18%): trainees 71, trainers 197, recent graduates and other members 44." [p.3 (Methods)] |

## S03 — Caretta-Weyer 2025

**Study ID:** S03 **Citation:** Caretta-Weyer 2025 (USA, Emergency Medicine) **Sample:** 8 EM residency programs (site leads)

**MMAT category (final adjudicated):** Category 4 — Quantitative descriptive **Met/5 (final adjudicated):** 3 **Quality category:** Moderate (3/5) **Disagreements adjudicated:** 1

| **Item** | **Item name** | **Reviewer A** | **Reviewer B** | **Final** | **Final justification (quoted evidence; adjudication rationale where ratings differed)** |
| --- | --- | --- | --- | --- | --- |
| S1 | Are there clear research questions? | Y | Y | Y | "Objective To develop and implement the 5 core components of CBME within 8 US emergency medicine (EM) residency programs and assess acceptability and feasibility." [p.57 (Abstract)] |
| S2 | Do the collected data allow to address the research questions? | Y | Y | Y | "We measured perceived feasibility and user acceptability of the CBME implementation process by surveying site leaders at the midpoint of year 5 of the grant funding period." [p.59 (Outcomes)] |
| 4.1 | Is the sampling strategy relevant to address the research question? | Y | Y | Y | "We implemented CBME in 8 EM residency programs, 6 of whom volunteered to participate from the onset of the RR project in 2019 and 2 others joined…" "Site leads were chosen from the residency program leadership teams, who served in this role for at…" [p.57 (Setting and Participants); p.59 (Outcomes)] |
| 4.2 | Is the sample representative of the target population? | N | CT | N | "Our findings regarding the implementation of this CBME intervention are limited by a small number of programs, similar size programs, geography, and program format. In addition, most of the programs were well-resourced and academically affiliated." [p.62 (Discussion)] Adjudication: Reviewer A: No (authors explicitly acknowledge non-representativeness); Reviewer B: Can't tell (cohort is self-selected/grant-funded but representativeness uncertain). Final adjudicated rating: No. The authors themselves explicitly state limited representativeness — small N (8), homogeneity, well-resourced, academic. Per MMAT, when authors clearly acknowledge non-representativeness, a No rating is appropriate. |
| 4.3 | Are the measurements appropriate? | CT | CT | CT | "The survey was drafted and reviewed by all site leads to optimize content and response process validity, and revised based on team feedback for clarity and content prior to collecting responses. The survey was not otherwise tested. The final survey…" [p.59 (Outcomes)] |
| 4.4 | Is the risk of nonresponse bias low? | Y | Y | Y | "All 8 site leads responded to the survey to assess perceptions regarding feasibility and acceptability." "The survey response rate was 100% (8 of 8)." [p.59 (Results); p.57 (Abstract)] |
| 4.5 | Is the statistical analysis appropriate to answer the research question? | Y | Y | Y | "We reported descriptive statistics, including ranges from the numerical responses, to open-ended questions asking about time and money. Means were often not feasible as sites responded with open-ended numbers often including their own program…" [p.59 (Analysis)] |

## S04 — Chan 2015

**Study ID:** S04 **Citation:** Chan 2015 (Canada, Emergency Medicine) **Sample:** 25 pre + 25 post McMAP end-of-rotation reports (1 program)

**MMAT category (final adjudicated):** Category 3 — Quantitative non-randomized **Met/5 (final adjudicated):** 3 **Quality category:** Moderate (3/5) **Disagreements adjudicated:** 0

| **Item** | **Item name** | **Reviewer A** | **Reviewer B** | **Final** | **Final justification (quoted evidence; adjudication rationale where ratings differed)** |
| --- | --- | --- | --- | --- | --- |
| S1 | Are there clear research questions? | CT | CT | CT | "In this report, we describe the development of the McMaster Modular Assessment Program (McMAP), a novel WBA system designed to integrate both quantitative and qualitative measures to generate robust reports on resident performance in the McMaster…" [p.900 (Problem)] |
| S2 | Do the collected data allow to address the research questions? | Y | Y | Y | "We compared CCERR scores of end-of-rotation reports from before and after the introduction of McMAP. We randomly selected 25 end-of-rotation reports for postgraduate year 1 and 2 residents from a pre-McMAP year (2010–2011, the year before McMAP was…" [p.902 (Outcomes)] |
| 3.1 | Are the participants representative of the target population? | CT | CT | CT | "We randomly selected 25 end-of-rotation reports for postgraduate year 1 and 2 residents from a pre-McMAP year (2010–2011, the year before McMAP was introduced) and an early McMAP year (2012–2013, the year after McMAP was piloted)." [p.902 (Outcomes)] |
| 3.2 | Are measurements appropriate regarding both the outcome and intervention (or exposure)? | Y | Y | Y | "To determine the efficacy of McMAP, we audited the quality of the end-of-rotation reports using the Completed Clinical Evaluation Report Rating (CCERR) tool. The CCERR tool, a nine-item scoring system to evaluate the quality of end-of-rotation…" [p.902 (Outcomes)] |
| 3.3 | Are there complete outcome data? | Y | Y | Y | "All 50 reports were independently scored by two investigators (T.C., J.S.) using the CCERR tool." [p.902 (Outcomes)] |
| 3.4 | Are the confounders accounted for in the design and analysis? | N | N | N | "We compared CCERR scores of end-of-rotation reports from before and after the introduction of McMAP… There was a doubling of median CCERR scores from the pre-McMAP year to the early McMAP year (13.8/45 [interquartile range = 11.3–15.8] versus…" [p.902 (Outcomes)] |
| 3.5 | During the study period, is the intervention administered (or exposure occurred) as intended? | Y | Y | Y | "In the first nine months, we gathered more than 4,000 data points for 15 residents in postgraduate years 1 and 2. These data points were 38% qualitative (written comments) and 62% quantitative (completed checklists, ratings of tasks or daily global…" [p.902 (Outcomes)] |

## S06 — Gauthier 2024

**Study ID:** S06 **Citation:** Gauthier 2024 (Canada, Internal Medicine) **Sample:** 20 IM residents (1 program)

**MMAT category (final adjudicated):** Category 1 — Qualitative **Met/5 (final adjudicated):** 5 **Quality category:** High (5/5) **Disagreements adjudicated:** 0

| **Item** | **Item name** | **Reviewer A** | **Reviewer B** | **Final** | **Final justification (quoted evidence; adjudication rationale where ratings differed)** |
| --- | --- | --- | --- | --- | --- |
| S1 | Are there clear research questions? | Y | Y | Y | "We sought to answer the following question: how do residents navigate the tensions between formative and summative purposes of WBA when deciding whether to initiate an assessment?" [p.480 (end of Introduction)] |
| S2 | Do the collected data allow to address the research questions? | Y | Y | Y | "We conducted 20 semi-structured interviews with internal medicine residents at Queen's University about the factors that influence their decision to seek or avoid WBA." [p.478 (Abstract — Approach)] |
| 1.1 | Is the qualitative approach appropriate to answer the research question? | Y | Y | Y | "We applied a constructivist grounded theory (CGT) approach to inform the development of an evidence informed model… we applied CGT to our study as it can build on past findings, frameworks, concepts, and theories in this field." [p.479-480 (Methods)] |
| 1.2 | Are the qualitative data collection methods adequate to address the research question? | Y | Y | Y | "Semi-structured interviews were conducted between November 2019 and February 2020 by a research associate with no association to the participants (HB). The interview protocol was based on a 12-question interview guide that was developed through…" [p.480 (Data collection)] |
| 1.3 | Are the findings adequately derived from the data? | Y | Y | Y | "We performed three levels of coding: open coding where each segment of text was assigned to a code; axial coding to identify connections between codes (categories) across transcripts; selective coding, to identify overarching patterns across…" [p.481 (Data analysis)] |
| 1.4 | Is the interpretation of results sufficiently substantiated by data? | Y | Y | Y | "Honestly, I need that number. A lot of the time the comments are not useful. They say, good job or great work or read more around your cases. And that is not helpful. A lot of the time it is to fulfill the requirements of the program in order to…" [p.481 (Results) and throughout pp.481-484] |
| 1.5 | Is there coherence between qualitative data sources, collection, analysis and interpretation? | Y | Y | Y | "To ensure trustworthiness of the data, as suggested by Nowell et al., we were very familiar with our data, member checked through summarizing the main discussion points at the end of each interview, triangulated the data, maintained an audit trail…" [p.480 (Data collection)] |

## S07 — Hauff 2014

**Study ID:** S07 **Citation:** Hauff 2014 (USA, Emergency Medicine) **Sample:** 28 EM interns from 2 cohorts (1 program)

**MMAT category (final adjudicated):** Category 4 — Quantitative descriptive **Met/5 (final adjudicated):** 4 **Quality category:** High (4/5) **Disagreements adjudicated:** 1

| **Item** | **Item name** | **Reviewer A** | **Reviewer B** | **Final** | **Final justification (quoted evidence; adjudication rationale where ratings differed)** |
| --- | --- | --- | --- | --- | --- |
| S1 | Are there clear research questions? | Y | Y | Y | "The objective of this study was to determine the feasibility and results of programmatic assessment of Level 1 milestones using multisource assessments for incoming EM interns in July." [p.694 (Abstract — Objectives); p.695 (end of Introduction)] |
| S2 | Do the collected data allow to address the research questions? | Y | Y | Y | "Assessment of milestones was done by four distinct methods: 1) the postgraduate orientation assessment (POA); 2) direct observation… 3) global milestone assessment… and 4) faculty assessment during procedural labs." [p.695 (Methods)] |
| 4.1 | Is the sampling strategy relevant to address the research question? | Y | Y | Y | "The study was conducted at a large EM residency program with 28 interns (July 2012 and 2013), representing 19 Liaison Committee on Medical Education–accredited medical schools." [p.695 (Study Setting and Population)] |
| 4.2 | Is the sample representative of the target population? | N | CT | N | "While the data for this study came from one residency program, that homogeneity is attenuated by the interns being observed across three different clinical sites by a diverse faculty body and that the interns come from a variety of medical schools…" [p.697 (Limitations)] Adjudication: Reviewer A: No (authors explicitly state representativeness limited); Reviewer B: Can't tell (single-program convenience sample). Final adjudicated rating: No. Authors explicitly state "We do not know… if our population is representative of interns at other programs." Single-program n=28 from one institution. Score N consistent with the author-acknowledged limitation. |
| 4.3 | Are the measurements appropriate? | Y | Y | Y | "This assessment has built validity evidence through careful design and scholarship, and the scores have been shown to correlate with our residents' performance." "The content validity of this instrument was derived from using the wording of the EM…" [p.695 (POA section); p.695 (Milestone-based CSA)] |
| 4.4 | Is the risk of nonresponse bias low? | Y | Y | Y | "The study was conducted at a large EM residency program with 28 interns (July 2012 and 2013)." [p.695 (Study Setting and Population)] |
| 4.5 | Is the statistical analysis appropriate to answer the research question? | Y | Y | Y | "Simple descriptive statistics are reported." [p.695 (Data Analysis)] |

## S08 — Lee 2022

**Study ID:** S08 **Citation:** Lee 2022 (Canada, Family Medicine) **Sample:** Archived fieldnotes from 1 FM program

**MMAT category (final adjudicated):** Category 3 — Quantitative non-randomized **Met/5 (final adjudicated):** 3 **Quality category:** Moderate (3/5) **Disagreements adjudicated:** 1

| **Item** | **Item name** | **Reviewer A** | **Reviewer B** | **Final** | **Final justification (quoted evidence; adjudication rationale where ratings differed)** |
| --- | --- | --- | --- | --- | --- |
| S1 | Are there clear research questions? | Y | Y | Y | "In competency-based medical education (CBME), should resident self-assessments be included in the array of evidence upon which summative progress decisions are made? We examined the congruence between self-assessments and preceptor assessments of…" [p.599 (Abstract — Background and Objectives)] |
| S2 | Do the collected data allow to address the research questions? | Y | Y | Y | "This was a retrospective observational cohort study using a learning analytics approach. The data source was archived formative workplace-based assessment forms (fieldnotes) stored in an online portfolio by family medicine residents and preceptors." [p.599 (Abstract — Methods)] |
| 3.1 | Are the participants representative of the target population? | CT | CT | CT | "We extracted data for this retrospective observational study from a database of archived formative workplace-based assessments… across three academic teaching sites over 3 academic years (2015-2016, 2016-2017, 2017-2018)." [p.600 (Setting and Participants)] |
| 3.2 | Are measurements appropriate regarding both the outcome and intervention (or exposure)? | Y | Y | Y | "'Progress level' in CBAS fieldnotes refers to resident progress in the development of skills and behaviors (competencies) important for a practicing physician, and is measured on a three-point scale: (1) 'Stop, Important Correction'… (2) 'In…" [p.600 (Outcomes Measured)] |
| 3.3 | Are there complete outcome data? | Y | Y | Y | "Over 3 academic years (2015-2016, 2016-2017, 2017-2018), 7,167 fieldnotes were entered at the three teaching sites included in this study… Of the total data set, 6,863 fieldnotes were examined for comparisons of resident-entered (n=1,229) and…" [p.601 (Results)] |
| 3.4 | Are the confounders accounted for in the design and analysis? | N | CT | N | "The biggest limitation is that we conducted analyses at the aggregate level… Resident self-reflection is subject to many inter- and intrapersonal factors such as gender dyad, age, and metacognitive ability; none of these were analyzed here…" [p.604 (Limitations)] Adjudication: Reviewer A: No (authors explicitly acknowledge no confounder adjustment); Reviewer B: Can't tell (no formal multivariable adjustment, but stratification by training year performed). Final adjudicated rating: No. Authors explicitly state confounders (gender dyad, age, metacognitive ability, preceptor training cohort) were NOT analyzed. Selection bias in self-entered fieldnotes is acknowledged by the authors but unadjusted, supporting the No rating. |
| 3.5 | During the study period, is the intervention administered (or exposure occurred) as intended? | Y | Y | Y | "The residency program expects that each resident receive at least one preceptor-entered fieldnote each week that the resident trains in family medicine clinics (approximately 40 per year); residents are encouraged to enter a similar number of…" [p.600 (Setting and Participants)] |

## S09 — McEwen 2015

**Study ID:** S09 **Citation:** McEwen 2015 (Canada, Family Medicine) **Sample:** Graduate survey n=32 (67%) + AA quotes + program stats (1 program)

**MMAT category (final adjudicated):** Category 5 — Mixed methods **Met/5 (final adjudicated):** 0 **Quality category:** Low (0/5) **Disagreements adjudicated:** 3

| **Item** | **Item name** | **Reviewer A** | **Reviewer B** | **Final** | **Final justification (quoted evidence; adjudication rationale where ratings differed)** |
| --- | --- | --- | --- | --- | --- |
| S1 | Are there clear research questions? | CT | CT | CT | "In this article, we describe the needs assessment, development, implementation, and continuing quality improvement processes that have shaped the Portfolio Assessment Support System (PASS) used in residency training in the Department of Family…" [p.1515 (Introduction)] |
| S2 | Do the collected data allow to address the research questions? | CT | CT | CT | "We use multiple measures to monitor the impact of PASS at the individual, programmatic, and institutional levels. At the individual level, we gather and examine learners' and faculty members' perspectives… At the programmatic level, we monitor…" [p.1519 (Program Evaluation)] |
| 5.1 | Is there an adequate rationale for using a mixed methods design to address the research question? | N | N | N | No explicit rationale for mixed methods is provided. [throughout — no rationale section] |
| 5.2 | Are the different components of the study effectively integrated to answer the research question? | CT | N | N | Survey data (e.g., "75% of Queen's family medicine residents (24/32) agreed or strongly agreed…") and AA interview quotes are presented sequentially without explicit integration, joint display, triangulation matrix, or analytic merging. [pp.1519-1520] Adjudication: Reviewer A: Can't tell; Reviewer B: No. Final adjudicated rating: No. The article presents quant survey results, qual AA quotes, and program statistics juxtaposed but with no formal integration mechanism. MMAT user guide indicates N for items where integration is clearly absent. |
| 5.3 | Are the outputs of the integration of qualitative and quantitative components adequately interpreted? | CT | N | N | Quantitative findings, qualitative quotes, and program statistics are presented sequentially as confirmatory evidence with limited cross-component interpretation; no joint display, no integrated analytic narrative. [p.1519-1521] Adjudication: Reviewer A: Can't tell; Reviewer B: No. Final adjudicated rating: No. There is no integrated interpretation of how quantitative survey findings and qualitative AA quotes converge or extend each other; each strand stands alone as evidence of program impact. |
| 5.4 | Are divergences and inconsistencies between quantitative and qualitative results adequately addressed? | N | CT | N | No discussion of any divergence between quantitative and qualitative findings is presented anywhere in the article; all findings are framed as confirmatory. [throughout] Adjudication: Reviewer A: No; Reviewer B: Can't tell. Final adjudicated rating: No. Both reviewers agree no divergences are addressed. A scored N because of the absence; B scored CT because absence of examination cannot be distinguished from absence of divergences. Per MMAT guidance, when divergences are clearly not addressed and the design is sequential without integration, score N. |
| 5.5 | Do the different components of the study adhere to the quality criteria of each tradition of the methods involved? | N | N | N | "The Graduate survey was piloted in the spring of 2013 at Queen's, and 67% (32/48) of the graduating family medicine residents completed it." "Interviews with AAs for an upcoming article suggest that they value…" [p.1519 (Individual level)] |

## S10 — O'Keeffe 2024

**Study ID:** S10 **Citation:** O'Keeffe 2024 (Ireland, Surgery (CST)) **Sample:** 114 national CST trainees (full cohort)

**MMAT category (final adjudicated):** Category 4 — Quantitative descriptive **Met/5 (final adjudicated):** 5 **Quality category:** High (5/5) **Disagreements adjudicated:** 0

| **Item** | **Item name** | **Reviewer A** | **Reviewer B** | **Final** | **Final justification (quoted evidence; adjudication rationale where ratings differed)** |
| --- | --- | --- | --- | --- | --- |
| S1 | Are there clear research questions? | Y | Y | Y | "The objective of this study was to evaluate the validity evidence supporting the use and interpretation of a multifaceted assessment system in the early years of surgical training." [p.1709 (Abstract — Objective)] |
| S2 | Do the collected data allow to address the research questions? | Y | Y | Y | "Data from all elements of the assessment process was evaluated using Messick's unified validity framework. Assessments were categorized as Workplace-based, Structured assessment performed in the academic center and Multiple Mini Interview." [p.1709 (Abstract — Design); p.1710 (Methods)] |
| 4.1 | Is the sampling strategy relevant to address the research question? | Y | Y | Y | "Data was collected from 2 cohorts of the entire population of postgraduate trainees nationally (N = 114)." [p.1709 (Abstract — Participants)] |
| 4.2 | Is the sample representative of the target population? | Y | Y | Y | "We included data from all trainees entering the training program, including those who had not completed all aspects of their training and assessment." [p.1711 (Study Setting and Subjects)] |
| 4.3 | Are the measurements appropriate? | Y | Y | Y | "For the technical skills assessments we used a combination of validated task-specific checklists and a widely published global rating tool, Objective Structured Assessment of Technical Skills (OSATS) to reduce the variance related to using the…" [p.1711 (Content section)] |
| 4.4 | Is the risk of nonresponse bias low? | Y | Y | Y | "Trainees with missing data due to missed assessments or early exit from the program were also included to present a comprehensive review of all data. For missing data in the generalizability study (G-study), we used the unbalanced random-effects…" [p.1713 (Internal Structure)] |
| 4.5 | Is the statistical analysis appropriate to answer the research question? | Y | Y | Y | "Generalizability theory was used to further analyses the reliability of this complex measurement system and identify the multiple sources of variability and potential sources of error using the urGENOVA software package… This method has been shown…" [p.1713 (Internal Structure)] |

## S11 — Park 2016

**Study ID:** S11 **Citation:** Park 2016 (USA, Internal Medicine) **Sample:** 142 IM residents (1 program, 1 year)

**MMAT category (final adjudicated):** Category 4 — Quantitative descriptive **Met/5 (final adjudicated):** 3 **Quality category:** Moderate (3/5) **Disagreements adjudicated:** 2

| **Item** | **Item name** | **Reviewer A** | **Reviewer B** | **Final** | **Final justification (quoted evidence; adjudication rationale where ratings differed)** |
| --- | --- | --- | --- | --- | --- |
| S1 | Are there clear research questions? | Y | Y | Y | "This study examines validity evidence of end-of-rotation evaluation scores that inform CCC's decisions for reporting milestones to the ACGME." [p.136 (Introduction)] |
| S2 | Do the collected data allow to address the research questions? | Y | Y | Y | "Data from 2,701 end-of-rotation evaluations measuring 21 out of 22 Internal Medicine milestones for 142 residents were analyzed (July 2013–June 2014)." [p.135 (Abstract — Approach)] |
| 4.1 | Is the sampling strategy relevant to address the research question? | Y | Y | Y | "Retrospective data from 2,701 end-of-rotation evaluations were used, from July 1, 2013, to June 30, 2014, across three postgraduate years (PGY)… all evaluations for all learners during this period were used." [p.136 (Methods — Data collection)] |
| 4.2 | Is the sample representative of the target population? | N | CT | N | "Empirical results from this study pertain to a single year of data collected at a single institution, based on milestones and rating levels that are institution specific." [p.142 (Discussion — limitations)] Adjudication: Reviewer A: No (authors explicitly state generalizability constrained); Reviewer B: Can't tell (single-program but complete within-program coverage). Final adjudicated rating: No. Authors explicitly acknowledge non-representativeness; single-institution single-year data; institution-specific milestones not generalizable to other IM residencies. The No rating is consistent with the authors’ own acknowledgment of limited generalizability. |
| 4.3 | Are the measurements appropriate? | Y | Y | Y | "Internal Medicine (IM) reportable milestones were used as global items with anchors corresponding to the milestone levels. A total of 21 out of 22 reportable IM milestones were used as items in the end-of-rotation evaluation forms…" "Each milestone…" [p.136 (End-of-rotation evaluation form)] |
| 4.4 | Is the risk of nonresponse bias low? | CT | Y | CT | "On average, raters took 89.91 days (SD = 91.60, Mdn = 46) to complete evaluation forms… Faculty took the longest (M = 111.03 days, SD = 95.27, Mdn = 91)…" [p.139 (Delays in scoring)] Adjudication: Reviewer A: Can't tell (substantial submission delays acknowledged, unbalanced ratings); Reviewer B: Yes (complete extraction of submitted evaluations). Final adjudicated rating: Can't tell. Although all submitted evaluations were extracted, the authors explicitly document substantial rater delays (~3 months) and unbalanced numbers of evaluations per resident, which leaves the completeness criterion uncertain. |
| 4.5 | Is the statistical analysis appropriate to answer the research question? | Y | Y | Y | "Generalizability analysis and higher order confirmatory factor analysis were used to examine the internal structure of ratings. Psychometric implications for combining evaluation scores using composite score reliability were examined." [p.135 (Abstract — Approach); pp.136-137 (Analysis)] |

## S12 — Park 2020

**Study ID:** S12 **Citation:** Park 2020 (USA, Internal Medicine) **Sample:** 34 IM residents over 3 years (1 program)

**MMAT category (final adjudicated):** Category 4 — Quantitative descriptive **Met/5 (final adjudicated):** 3 **Quality category:** Moderate (3/5) **Disagreements adjudicated:** 2

| **Item** | **Item name** | **Reviewer A** | **Reviewer B** | **Final** | **Final justification (quoted evidence; adjudication rationale where ratings differed)** |
| --- | --- | --- | --- | --- | --- |
| S1 | Are there clear research questions? | Y | Y | Y | "This article describes development, administration, and psychometric analyses of a learner analytics system to resolve challenges in implementation of milestones by introducing the Scoring Grid Model, operationalized in an internal medicine (IM)…" [p.599 (Abstract — Purpose)] |
| S2 | Do the collected data allow to address the research questions? | Y | Y | Y | "A 3-year longitudinal cohort of 34 residents at the University of Illinois at Chicago College of Medicine began using this learner analytics system, from entry (July 2013) to graduation (June 2016). Scores from 23 assessments used throughout the…" [p.599 (Abstract — Method)] |
| 4.1 | Is the sampling strategy relevant to address the research question? | Y | Y | Y | "The first cohort of IM residents (n = 34) who were assessed using the Scoring Grid Model graduated from the program on June 2016. Milestone levels reported to the ACGME for this cohort of residents were downloaded for the 5 required milestone level…" [p.601 (Analysis)] |
| 4.2 | Is the sample representative of the target population? | N | CT | N | "This study was based on data from a single residency program… Future studies are needed to generalize the applicability of the Scoring Grid Model to other institutions and residency programs that may use varying weights to reflect curricular…" [p.608 (Discussion)] Adjudication: Reviewer A: No (authors explicitly limited to single program); Reviewer B: Can't tell (single-institution sample). Final adjudicated rating: No. Authors explicitly state limited generalizability beyond single program (UI-COM IM residency, n=34). Applied consistently with S07/S11. |
| 4.3 | Are the measurements appropriate? | Y | Y | Y | "The IM residency program identified 23 assessments (in addition to CCC review) used throughout the 3-year training period… All assessments were standardized to scores on a 9-point scale, following the same 9-point scoring level for the milestones." [p.601-602 (Results — Content)] |
| 4.4 | Is the risk of nonresponse bias low? | CT | Y | CT | "By training year, the Φ coefficients for reliability were .81, 0.46, and .76 for PGY-1, PGY-2, and PGY-3, respectively. Reliability dropped during PGY-2 due to a lower number of evaluations collected per learner." [p.606 (Reliability)] Adjudication: Reviewer A: Can't tell (substantial unbalance: PGY-2 only ~8 evaluations vs ~27 for PGY-1); Reviewer B: Yes (full cohort followed, mandated assessments). Final adjudicated rating: Can't tell. The Φ reliability dropped to 0.46 in PGY-2 due to substantially lower number of evaluations — unbalanced rater submission introduces a missingness/non-response concern that is acknowledged but not analyzed, so the criterion remains uncertain. |
| 4.5 | Is the statistical analysis appropriate to answer the research question? | Y | Y | Y | "We used standardized β coefficients from mixed-effects regression controlling for clustering over time to examine associations between assessment scores and the milestone level… Generalizability theory was used to examine the reliability of…" [p.601 (Analysis)] |

## S13 — Park 2021

**Study ID:** S13 **Citation:** Park 2021 (USA, Family Medicine) **Sample:** 3,872 FM residents in 514 ACGME programs (national)

**MMAT category (final adjudicated):** Category 4 — Quantitative descriptive **Met/5 (final adjudicated):** 5 **Quality category:** High (5/5) **Disagreements adjudicated:** 0

| **Item** | **Item name** | **Reviewer A** | **Reviewer B** | **Final** | **Final justification (quoted evidence; adjudication rationale where ratings differed)** |
| --- | --- | --- | --- | --- | --- |
| S1 | Are there clear research questions? | Y | Y | Y | "To investigate the reliability of learning trajectories and patterns of learning progression that can support meaningful intervention and remediation for residents." [p.1 (Abstract — Objective)] |
| S2 | Do the collected data allow to address the research questions? | Y | Y | Y | "This national retrospective cohort study included Milestones data from residents in family medicine, representing 6 semi-annual reporting periods from July 2016 to June 2019." [p.1 (Abstract — Design); p.3 (Methods)] |
| 4.1 | Is the sampling strategy relevant to address the research question? | Y | Y | Y | "We used national cohort data from 514 residency programs and 3872 residents in family medicine who reported their Milestones data to the ACGME between 2016 and 2019." [p.3 (Methods — Study Design, Setting, and Participants)] |
| 4.2 | Is the sample representative of the target population? | Y | Y | Y | "This study includes national data belonging to family medicine residents from entry to graduation who began training in 2016 and graduated in 2019. As such, we include all learners from the national database who trained in family medicine during…" [p.5 (Bias section)] |
| 4.3 | Are the measurements appropriate? | Y | Y | Y | "Family medicine is a 3-year training program with 22 subcompetencies across the 6 ACGME Core Competencies… The Milestones data are on a 10-point scale between level 1 and level 5 in 0.50-unit intervals… Validity evidence supporting Milestones…" [p.3 (Methods — Family Medicine Milestones)] |
| 4.4 | Is the risk of nonresponse bias low? | Y | Y | Y | "This study found that approximately 10% of learners did not graduate in family medicine within the 3-year training period, warranting further study of the reasons why residents did not graduate in time…" [p.11 (Limitations); p.5 (Bias)] |
| 4.5 | Is the statistical analysis appropriate to answer the research question? | Y | Y | Y | "Reliability metrics (GRR and GCR) were estimated following the specification in Willett and in Hertzog et al… For the GMMs, we fit as many as 10 learning trajectories and used model fit indices (information criteria and classification indices) to…" [p.5 (Statistical Analysis)] |

## S14 — Paternotte 2024

**Study ID:** S14 **Citation:** Paternotte 2024 (Netherlands/Canada, OBGYN) **Sample:** 1 main auto-ethnographer + reflective discussions

**MMAT category (final adjudicated):** Category 1 — Qualitative **Met/5 (final adjudicated):** 1 **Quality category:** Low (1/5) **Disagreements adjudicated:** 3

| **Item** | **Item name** | **Reviewer A** | **Reviewer B** | **Final** | **Final justification (quoted evidence; adjudication rationale where ratings differed)** |
| --- | --- | --- | --- | --- | --- |
| S1 | Are there clear research questions? | Y | Y | Y | "The research questions for this study are as follows: what are the differences in program structure and assessment program in Obstetrics and Gynecology postgraduate medical education in the Netherlands and Canada? And how does this impact the…" [p.1 (Abstract — Materials and methods)] |
| S2 | Do the collected data allow to address the research questions? | CT | Y | CT | "We compared both countries' postgraduate educational blueprints and used an auto-ethnographic method to gain insight in the effects of training program structure and assessment methods on how trainees work." [p.1 (Abstract); p.2 (Materials and methods)] Adjudication: Reviewer A: Can't tell (comparative breadth constrained — primarily one trainee's elective experience plus colleague discussions); Reviewer B: Yes (blueprint comparison + EP's elective + reflections map to research questions). Final adjudicated rating: Can't tell. Auto-ethnography with one main auto-ethnographer (EP) plus reflective sessions only partially addresses the comparative aim across the two national systems, leaving the criterion uncertain. |
| 1.1 | Is the qualitative approach appropriate to answer the research question? | Y | Y | Y | "This study is grounded in auto-ethnography, a qualitative research methodology in which the author uses writing and self-reflection to probe personal experiences, thereby deriving broader sociocultural meaning and understanding." [p.2 (Materials and methods)] |
| 1.2 | Are the qualitative data collection methods adequate to address the research question? | CT | CT | CT | "EP's experiences as an elective OBGYN postgraduate trainee were discussed with the other researchers… These findings were then discussed with gynecologists and postgraduate trainees of both the Netherlands and British Columbia, Canada. Extensive…" [p.3 (Data collection)] |
| 1.3 | Are the findings adequately derived from the data? | CT | CT | CT | "Reflection: Postgraduate medical education in Canada and the Netherlands share the same philosophy… Despite these similarities, there are a few key differences in the curricula and assessment methods experienced by EP and discussed with the…" [p.5 (Reflection)] |
| 1.4 | Is the interpretation of results sufficiently substantiated by data? | Y | CT | CT | Article relies primarily on EP's personal reflections and curricular blueprint comparison; few participant quotations beyond the supervisor quote ("Competency by design is still a bit out of reality of daily clinical education.") [p.5-6 (Reflection); Tables 1-3] Adjudication: Reviewer A: Yes (extensive blueprint comparison + some quotations); Reviewer B: Can't tell (sparse verbatim data). Final adjudicated rating: Can't tell. While curricular blueprints are documented, interpretive claims about culture and entrustment are sparsely supported with verbatim qualitative data. Conservative scoring per MMAT guidance preserves uncertainty. |
| 1.5 | Is there coherence between qualitative data sources, collection, analysis and interpretation? | CT | Y | CT | "A limitation of this study is that not all perspectives could be captured. There may be opinions and experiences of trainees, teachers, and supervisors that were missed. Other limitations include the inherent subjectivity of the ethnography method…" [p.7 (Limitations)] Adjudication: Reviewer A: Can't tell (mix of personal reflection + blueprint comparison not fully integrated analytically); Reviewer B: Yes (auto-ethnographic framing coherent). Final adjudicated rating: Can't tell. The hybrid of formal blueprint review + personal reflection is partially coherent with auto-ethnographic methodology, but analytic detail and integration between the two strands is insufficiently described to verify, supporting the Can’t tell rating. |

## S15 — Perry 2018

**Study ID:** S15 **Citation:** Perry 2018 (USA, Emergency Medicine) **Sample:** Mapping of assessment modalities (1 program)

**MMAT category (final adjudicated):** Category 4 — Quantitative descriptive **Met/5 (final adjudicated):** 0 **Quality category:** Low (0/5) **Disagreements adjudicated:** 0

| **Item** | **Item name** | **Reviewer A** | **Reviewer B** | **Final** | **Final justification (quoted evidence; adjudication rationale where ratings differed)** |
| --- | --- | --- | --- | --- | --- |
| S1 | Are there clear research questions? | CT | CT | CT | "Objective We developed a system of programmatic assessment (PA) to identify competency progression (summative) and assessment for learning to assist residents in their formative development." [p.84 (Abstract)] |
| S2 | Do the collected data allow to address the research questions? | CT | CT | CT | "Through the PA, the 6 competency domains are assessed through multiple modalities: patient care (22 different assessments), professionalism (18), systems-based practice (17), interprofessional and communication skills (16), medical knowledge (11)…" [p.84 (Abstract — Results)] |
| 4.1 | Is the sampling strategy relevant to address the research question? | CT | CT | CT | "We implemented our programmatic assessment in a 4-year emergency medicine residency with 16 residents per year at a university hospital and 2 affiliated community hospitals." [p.84 (Methods — Setting)] |
| 4.2 | Is the sample representative of the target population? | N | N | N | "There are several limitations to this study. First, it was a single program, which limited generalizability." [p.89 (Discussion)] |
| 4.3 | Are the measurements appropriate? | CT | CT | CT | "The number of modalities for each competency domain was assessed." "The system was accepted by the residents, as demonstrated by our ACGME resident survey rating the program at or above the national mean for most of the elements that compose the…" [p.85 (Methods); p.89 (Results)] |
| 4.4 | Is the risk of nonresponse bias low? | CT | CT | CT | No specific reporting of response rates or nonresponse handling for the cited ACGME survey. [p.89 (Results)] |
| 4.5 | Is the statistical analysis appropriate to answer the research question? | CT | CT | CT | "From a practical perspective, through rigorous programmatic assessment, all of our residents were successfully progressing through the program." [p.89 (Results)] |

## S16 — Rich 2020

**Study ID:** S16 **Citation:** Rich 2020 (Canada, Emergency Medicine) **Sample:** Multi-stakeholder interviews (1 EM program)

**MMAT category (final adjudicated):** Category 1 — Qualitative **Met/5 (final adjudicated):** 5 **Quality category:** High (5/5) **Disagreements adjudicated:** 0

| **Item** | **Item name** | **Reviewer A** | **Reviewer B** | **Final** | **Final justification (quoted evidence; adjudication rationale where ratings differed)** |
| --- | --- | --- | --- | --- | --- |
| S1 | Are there clear research questions? | Y | Y | Y | "The dual purposes of this research were to: (1) illustrate a model of programmatic assessment in action and (2) identify strengths and challenges with the approach to operationalization." [p.1088 (end of Introduction)] |
| S2 | Do the collected data allow to address the research questions? | Y | Y | Y | "Qualitative data were collected from residents, faculty, and program leadership via semi-structured group and individual interviews conducted at nine months post-CBME implementation." [p.1087 (Abstract — Method)] |
| 1.1 | Is the qualitative approach appropriate to answer the research question? | Y | Y | Y | "In this study, the authors adopted a qualitative, interpretive case study design to better understand how a competency-based residency training program is approaching the operationalization of programmatic assessment." "Interpretive case studies…" [p.1088 (Methods — Study design)] |
| 1.2 | Are the qualitative data collection methods adequate to address the research question? | Y | Y | Y | "All program stakeholders—including the program leaders, residents, and faculty—were invited, via email, to participate in either an individual or group interview… Interviews continued until no new insights emerged from conversations with…" [p.1089-1090 (Data collection)] |
| 1.3 | Are the findings adequately derived from the data? | Y | Y | Y | "Data were analyzed using an abductive approach… NVivo software was used to annotate and code the dataset… In the third round of reading, the data assigned to each code was checked for internal homogeneity and external heterogeneity… Systems…" [p.1090 (Data analysis)] |
| 1.4 | Is the interpretation of results sufficiently substantiated by data? | Y | Y | Y | "I'm happy to give a pixel about something I've observed, and I'll give a bit of constructive feedback around the interaction, but sometimes the larger issues that are buzzing in my head are not represented in that because I'm uncomfortable with my…" [p.1091 (Results)] |
| 1.5 | Is there coherence between qualitative data sources, collection, analysis and interpretation? | Y | Y | Y | "Upon completion of the draft manuscript, a select sample of key participant informants were invited to review the draft and to provide feedback. Salient feedback was subsequently incorporated into the manuscript." [p.1090 (Data analysis); p.1094 (Limitations)] |

## S17 — Rich 2022

**Study ID:** S17 **Citation:** Rich 2022 (Canada, Multi-specialty) **Sample:** 4 specialty programs (focus groups)

**MMAT category (final adjudicated):** Category 1 — Qualitative **Met/5 (final adjudicated):** 5 **Quality category:** High (5/5) **Disagreements adjudicated:** 0

| **Item** | **Item name** | **Reviewer A** | **Reviewer B** | **Final** | **Final justification (quoted evidence; adjudication rationale where ratings differed)** |
| --- | --- | --- | --- | --- | --- |
| S1 | Are there clear research questions? | Y | Y | Y | "Specifically, our research questions were as follows: (1) Does our previously developed model of programmatic assessment (PA), which is based on the operationalization of PA within one EM program at Queen's University, reflect the models being…" [p.2 (Introduction)] |
| S2 | Do the collected data allow to address the research questions? | Y | Y | Y | "We conducted virtual focus groups with program leaders from four residency training programs from different disciplines (internal medicine, emergency medicine, neurology, and rheumatology) and institutions." [p.1 (Abstract)] |
| 1.1 | Is the qualitative approach appropriate to answer the research question? | Y | Y | Y | "We adopted a qualitative, multiple (multi-)case study design to model, interpret, and discuss how resident-level variables, specifically engagement in learning and performance (strength), influence the functioning of four programs of assessment… We…" [p.3-4 (Methods — Study Design)] |
| 1.2 | Are the qualitative data collection methods adequate to address the research question? | Y | Y | Y | "We conducted separate 90 min virtual focus groups with Competence Committee members from each specialty training program, using a set of pre-determined questions and two moderators (JVR and AKH)… During focus groups, one moderator (JVR) focused on…" [p.4 (Data Collection)] |
| 1.3 | Are the findings adequately derived from the data? | Y | Y | Y | "In alignment with semi-structured qualitative research, data analysis began with data collection. As the moderators (JVR and AKH) inductively picked-up on reoccurring and discrepant insights between cases (between archetypes and specialty…" [p.5 (Data Analysis)] |
| 1.4 | Is the interpretation of results sufficiently substantiated by data? | Y | Y | Y | "Participants explained how 'other systems get activated' when a resident is 'flagged' or thought to be 'in trouble'." Plus extensive direct quotations: "lots of assessment data", "engage more", "reading between the lines", "backend hallway…" [p.6-9 (Results)] |
| 1.5 | Is there coherence between qualitative data sources, collection, analysis and interpretation? | Y | Y | Y | "We chose an interpretive, multi-case study given its utility for building understanding of how educational initiatives work (or fail to work as intended) across contexts, in order to share lessons learned about implementation." [p.4 (Methods — Study Design); throughout] |

## S18 — Ross 2023

**Study ID:** S18 **Citation:** Ross 2023 (Canada, Family Medicine) **Sample:** Institutional audit (4 programs) + 15 published evaluation studies

**MMAT category (final adjudicated):** Category 5 — Mixed methods **Met/5 (final adjudicated):** 2 **Quality category:** Low (2/5) **Disagreements adjudicated:** 1

| **Item** | **Item name** | **Reviewer A** | **Reviewer B** | **Final** | **Final justification (quoted evidence; adjudication rationale where ratings differed)** |
| --- | --- | --- | --- | --- | --- |
| S1 | Are there clear research questions? | CT | CT | CT | "In this article, we describe the development and implementation of CBME in family medicine postgraduate training in Canada, with an emphasis on the approach to programmatic assessment. We present a meta-evaluation of findings that support the…" [p.188 (Introduction)] Adjudication: Both A and B rated CT. Final adjudicated rating: Can't tell. The aim is descriptive (description + meta-evaluation), not framed as a focused research question. |
| S2 | Do the collected data allow to address the research questions? | Y | Y | Y | "Evaluation data for CRAFT come from multiple sources, making a meta-evaluation an appropriate approach. Using a theory-led, outcomes-focused program evaluation framework, our meta-evaluation focused on the assumptions of CRAFT using 2 sources of…" [p.193 (Meta-Evaluation of CRAFT)] Adjudication: Both A and B rated Y. Final adjudicated rating: Yes. Institutional audit data + 15 published evaluation studies aligned with 3 CRAFT assumptions provide data sufficient to address the descriptive aim. |
| 5.1 | Is there an adequate rationale for using a mixed methods design to address the research question? | CT | N/A (B classified as Cat 1) | CT | "Evaluation data for CRAFT come from multiple sources, making a meta-evaluation an appropriate approach." [p.193] Adjudication: A rated CT (rationale not explicit). Final adjudicated rating: Can't tell (carry forward A's rating for Cat 5 framework). The rationale "multiple sources making meta-evaluation appropriate" is implicit; no explicit articulation of why mixed methods integrate qualitative content analysis with quantitative audit. Adjudicator note: the study was classified as Category 5 (overriding Reviewer B’s Category 1 assignment), and the five mixed-methods items were then rated under that category. |
| 5.2 | Are the different components of the study effectively integrated to answer the research question? | Y | N/A (B classified as Cat 1) | Y | "The data were analyzed using qualitative content analysis to summarize the existing evaluation findings and align those findings with 3 key assumptions of CRAFT." [p.194] Adjudication: A rated Y. Final adjudicated rating: Yes. The institutional audit and qualitative content analysis are integrated through the 3 CRAFT assumptions framework — both strands aligned to the same analytic framework. |
| 5.3 | Are the outputs of the integration of qualitative and quantitative components adequately interpreted? | Y | N/A (B classified as Cat 1) | Y | "While the findings from our meta-evaluation support the belief that CRAFT is having a beneficial effect on family medicine residency training, more findings are needed from large-scale studies across multiple programs, as well as long-term outcomes…" [p.195] Adjudication: A rated Y. Final adjudicated rating: Yes. Outputs of integration are interpreted in concluding paragraphs, identifying convergent support across audit data and published evaluation evidence. |
| 5.4 | Are divergences and inconsistencies between quantitative and qualitative results adequately addressed? | CT | N/A (B classified as Cat 1) | CT | No explicit examination of divergences between the institutional audit and qualitative content analysis findings is reported. [p.193-195] Adjudication: A rated CT. Final adjudicated rating: Can't tell. Limited discussion of divergences; findings are framed as convergent support for CRAFT, but no formal analysis of disconfirming evidence. |
| 5.5 | Do the different components of the study adhere to the quality criteria of each tradition of the methods involved? | CT | N/A (B classified as Cat 1) | CT | "The published evaluation data for the meta-evaluation came from an existing repository of 13 publications… One author (S.R.) then searched MEDLINE, EMBASE, and PsycInfo… The same author (S.R.) scanned the titles and abstracts of the 35 results and…" [p.194] Adjudication: A rated CT. Final adjudicated rating: Can't tell. Quantitative institutional audit is convenience sample of 4 programs; qualitative literature search is single-author screening with no PRISMA, no quality appraisal, and heavy reliance on the author group's own publications. Each strand has methodological limitations. |

## S19 — Schultz 2016

**Study ID:** S19 **Citation:** Schultz 2016 (Canada, Family Medicine) **Sample:** 24,000 data points / 150 residents / 8 of 350 extensions (1 program)

**MMAT category (final adjudicated):** Category 4 — Quantitative descriptive **Met/5 (final adjudicated):** 0 **Quality category:** Low (0/5) **Disagreements adjudicated:** 4

| **Item** | **Item name** | **Reviewer A** | **Reviewer B** | **Final** | **Final justification (quoted evidence; adjudication rationale where ratings differed)** |
| --- | --- | --- | --- | --- | --- |
| S1 | Are there clear research questions? | CT | N | CT | "This report will outline the steps we took to implement CBME in our program, highlighting facilitating factors and processes or steps that in retrospect we wish we had taken." [p.685 (Problem)] Adjudication: Reviewer A: Can't tell (Innovation Report; aims descriptive, not framed as research question); Reviewer B: No (no research question). Final adjudicated rating: Can't tell. As an Innovation Report, the article was not designed as research and lacks a focused research question. Per MMAT guidance, when the criterion is not directly applicable to the design, a Can’t tell rating is more appropriate than No; the rating reflects design inapplicability rather than methodological deficiency. |
| S2 | Do the collected data allow to address the research questions? | CT | N | CT | "Early outcomes are encouraging. Residents are being directly observed more often with increased documented feedback about performance based on explicit competency standards (24,000 data points for 150 residents from 2013 to 2015)." [p.685 (Outcomes)] Adjudication: Reviewer A: Can't tell; Reviewer B: No. Final adjudicated rating: Can't tell. Limited descriptive outcome data (counts of data points, 8/350 extensions, 4 dismissals) only partially address the implementation report's claims; without a formal study design, whether the data "address the research question" cannot be properly judged. |
| 4.1 | Is the sampling strategy relevant to address the research question? | CT | N | CT | "From 2013 to 2015 we have gathered over 24,000 data points for our 150 residents." [p.687 (Outcomes)] Adjudication: Reviewer A: Can't tell (sampling implicit, whole-program data); Reviewer B: No (no sampling strategy described). Final adjudicated rating: Can't tell. Sampling is implicit (entire program data extraction); appropriate for description but the absence of formal sampling strategy in an Innovation Report does not equate to inappropriate sampling — CT preserves the ambiguity. |
| 4.2 | Is the sample representative of the target population? | N | N | N | No representativeness analysis presented; single-program data only. [throughout] |
| 4.3 | Are the measurements appropriate? | CT | N | CT | "Each resident has an academic advisor (AA) with whom the resident meets every four months to review all assessment data, identifying patterns of performance, red flags, and competency development trajectory…" [p.687 (Steps 6 and 7)] Adjudication: Reviewer A: Can't tell; Reviewer B: No. Final adjudicated rating: Can't tell. Field notes and EPA-FNs are used as program "data points" but no psychometric measurement is conducted; the article does not present validated outcome measures. Score CT because measurements are not the focus of an Innovation Report. |
| 4.4 | Is the risk of nonresponse bias low? | CT | CT | CT | No response rate or non-response analysis described. [throughout] |
| 4.5 | Is the statistical analysis appropriate to answer the research question? | N | N | N | "Following review by our Resident Assessment Committee… an extension of training has occurred for 8 of about 350 residents from 2010 to 2015 for a total of 19 extra training blocks." [p.689 (Outcomes)] |

## S20 — Schut 2018

**Study ID:** S20 **Citation:** Schut 2018 (NL/Canada/UK, Family Medicine + Other) **Sample:** 26 learners across 5 programs in 3 countries

**MMAT category (final adjudicated):** Category 1 — Qualitative **Met/5 (final adjudicated):** 5 **Quality category:** High (5/5) **Disagreements adjudicated:** 0

| **Item** | **Item name** | **Reviewer A** | **Reviewer B** | **Final** | **Final justification (quoted evidence; adjudication rationale where ratings differed)** |
| --- | --- | --- | --- | --- | --- |
| S1 | Are there clear research questions? | Y | Y | Y | "Therefore, the current study aims to gain more insight into how assessment stakes are perceived by learners and which factors influence learners' perceptions." [p.655 (Introduction)] |
| S2 | Do the collected data allow to address the research questions? | Y | Y | Y | "Twenty-six learners were interviewed from three different countries and five different programs, ranging from undergraduate to postgraduate medical education. The interviews explored learners' experience with and perception of assessment stakes." [p.654 (Abstract — Methods)] |
| 1.1 | Is the qualitative approach appropriate to answer the research question? | Y | Y | Y | "We used an open and qualitative approach to data gathering and analyses, inspired by constructivist grounded theory." [p.655 (Methods — Sample)] |
| 1.2 | Are the qualitative data collection methods adequate to address the research question? | Y | Y | Y | "A total of 26 respondents participated in individual, semi-structured interviews. Open-ended questions were posed by one interviewer (SS), who asked participants to describe their assessment experiences… Interviews and analyses were conducted…" [p.656-657 (Data collection)] |
| 1.3 | Are the findings adequately derived from the data? | Y | Y | Y | "Interview data were analyzed using a constant comparative approach. Independent analysis of the first four transcripts using an open coding strategy was carried out by SS and SH. During this process, coding results and relations between codes were…" [p.657 (Data analysis)] |
| 1.4 | Is the interpretation of results sufficiently substantiated by data? | Y | Y | Y | "You have more control over the assessment [initiating a Mini-CEX] and then you can focus the assessment to what is important to you. You can tailor it to what you need at that moment. That makes it low-stake and more meaningful. (C2)" [p.658 (Results) and throughout pp.657-660] |
| 1.5 | Is there coherence between qualitative data sources, collection, analysis and interpretation? | Y | Y | Y | "We acknowledge that data in this study are co-constructed by interactions with the participants, as are the interpretations and meaning we gave to these data. To prevent biases as much as possible, we brought together a multidisciplinary research…" [p.657 (Reflexivity)] |

## S21 — Woodworth 2024

**Study ID:** S21 **Citation:** Woodworth 2024 (USA, Anesthesiology) **Sample:** 49,240 assessments across 7 academic anesthesiology programs (24 months)

**MMAT category (final adjudicated):** Category 4 — Quantitative descriptive **Met/5 (final adjudicated):** 3 **Quality category:** Moderate (3/5) **Disagreements adjudicated:** 0

| **Item** | **Item name** | **Reviewer A** | **Reviewer B** | **Final** | **Final justification (quoted evidence; adjudication rationale where ratings differed)** |
| --- | --- | --- | --- | --- | --- |
| S1 | Are there clear research questions? | Y | Y | Y | "The aims of this study were to (1) remap the 2018 EPA and procedural skills assessments to the revised ACGME Anesthesiology Milestones 2.0, (2) develop new assessments that combined with the original assessments to create a system of assessment…" [p.1081 (Abstract — Background)] |
| S2 | Do the collected data allow to address the research questions? | Y | Y | Y | "Following a 24-month pilot at 7 institutions, the number of EPA and procedural skill assessments and mean scores were computed at the end of the academic year. Milestone achievement and subcompetency data for assessments from a single institution…" [p.1081 (Abstract — Methods)] |
| 4.1 | Is the sampling strategy relevant to address the research question? | Y | Y | Y | "Seven programs agreed to participate: Oregon Health & Science University, University of Florida Jacksonville, University of Wisconsin, Augusta University, University of Minnesota, University of North Carolina, and Beth Israel Deaconess Medical…" [p.1083 (Methods); p.1085 (Results)] |
| 4.2 | Is the sample representative of the target population? | CT | CT | CT | "Seven programs agreed to participate: Oregon Health & Science University, University of Florida Jacksonville, University of Wisconsin, Augusta University, University of Minnesota, University of North Carolina, and Beth Israel Deaconess Medical…" [p.1083 (Methods)] |
| 4.3 | Are the measurements appropriate? | Y | Y | Y | "A modified iterative Delphi process was used to reach consensus on new assessments." "All assessments were mapped to milestones and then implemented in a mobile app and web-based application from myTIPreport." [p.1083 (Methods)] |
| 4.4 | Is the risk of nonresponse bias low? | CT | CT | CT | "A major limitation of this study was missing assessments. Lower milestone achievement and subcompetency scores computed in the assessment system compared to those of the CCC were largely attributable to missing NTSAs and OSCEs, which were only…" [p.1090 (Discussion — limitations)] |
| 4.5 | Is the statistical analysis appropriate to answer the research question? | Y | Y | Y | "Analyses were performed with Microsoft Excel, SPSS statistical analysis software, version 29.0, and RStudio… A series of 4 linear regression models were created to compare mean scores between trainees at different levels of training… Two-sample…" [p.1085 (Statistical Analysis)] |

# 5. Disagreement log

All item-level (and one category-level) disagreements between Reviewer A and Reviewer B, together with the FINAL rating and adjudication rationale.

| **Study ID** | **First Author Year** | **Item code** | **Item name** | **Reviewer A** | **Reviewer B** | **Final** | **Adjudication rationale** |
| --- | --- | --- | --- | --- | --- | --- | --- |
| S18 | Ross 2023 | CATEGORY | MMAT Category Assignment | Category 5 — Mixed methods | Category 1 — Qualitative (qualitative content-analysis-based meta-evaluation) | Category 5 — Mixed methods | S18: Reviewer A assigned Category 5 (mixed methods); Reviewer B assigned Category 1 (qualitative). The adjudicator’s final assignment was Category 5. The article integrates a quantitative institutional audit (Table 2: mean field notes, mean assessments, mean progress reviews, % needing extension across 4 programs) with qualitative content analysis of 15 published evaluation studies (Appendix 1) aligned to 3 CRAFT assumptions. Both empirical strands are present and integrated under the meta-evaluation framework — meets MMAT mixed-methods criteria. Reviewer B’s Category 1 assignment did not account for the quantitative audit strand. |
| S02 | Ashman 2025 | 5.1 | Is there an adequate rationale for using a mixed methods design to address the research question? | CT | Y | Y | Reviewer A: Can't tell (rationale not made explicit beyond design statement); Reviewer B: Yes (sequential explanatory rationale stated). Final adjudicated rating: Yes. The article explicitly identifies the design (sequential explanatory: quantitative TIMS/survey results informed focus-group questions) and links it to the Core Components Framework (Van Melle et al. 2019). MMAT 5.1 asks for an adequate rationale for mixed methods; the sequential explanatory logic (combining portfolio metrics with stakeholder perspectives to explain implementation outcomes) constitutes an adequate rationale. |
| S03 | Caretta-Weyer 2025 | 4.2 | Is the sample representative of the target population? | N | CT | N | Reviewer A: No (authors explicitly acknowledge non-representativeness); Reviewer B: Can't tell (cohort is self-selected/grant-funded but representativeness uncertain). Final adjudicated rating: No. The authors themselves explicitly state limited representativeness — small N (8), homogeneity, well-resourced, academic. Per MMAT, when authors clearly acknowledge non-representativeness, N is appropriate. |
| S07 | Hauff 2014 | 4.2 | Is the sample representative of the target population? | N | CT | N | Reviewer A: No (authors explicitly state representativeness limited); Reviewer B: Can't tell (single-program convenience sample). Final adjudicated rating: No. Authors explicitly state "We do not know… if our population is representative of interns at other programs." Single-program n=28 from one institution. Score N consistent with the author-acknowledged limitation. |
| S08 | Lee 2022 | 3.4 | Are the confounders accounted for in the design and analysis? | N | CT | N | Reviewer A: No (authors explicitly acknowledge no confounder adjustment); Reviewer B: Can't tell (no formal multivariable adjustment, but stratification by training year performed). Final adjudicated rating: No. Authors explicitly state confounders (gender dyad, age, metacognitive ability, preceptor training cohort) were NOT analyzed. Selection bias in self-entered fieldnotes is also unaddressed. Score N. |
| S09 | McEwen 2015 | 5.2 | Are the different components of the study effectively integrated to answer the research question? | CT | N | N | Reviewer A: Can't tell; Reviewer B: No. Final adjudicated rating: No. The article presents quant survey results, qual AA quotes, and program statistics juxtaposed but with no formal integration mechanism. MMAT user guide indicates N for items where integration is clearly absent. |
| S09 | McEwen 2015 | 5.3 | Are the outputs of the integration of qualitative and quantitative components adequately interpreted? | CT | N | N | Reviewer A: Can't tell; Reviewer B: No. Final adjudicated rating: No. There is no integrated interpretation of how quantitative survey findings and qualitative AA quotes converge or extend each other; each strand stands alone as evidence of program impact. |
| S09 | McEwen 2015 | 5.4 | Are divergences and inconsistencies between quantitative and qualitative results adequately addressed? | N | CT | N | Reviewer A: No; Reviewer B: Can't tell. Final adjudicated rating: No. Both reviewers agree no divergences are addressed. A scored N because of the absence; B scored CT because absence of examination cannot be distinguished from absence of divergences. Per MMAT guidance, when divergences are clearly not addressed and the design is sequential without integration, score N. |
| S11 | Park 2016 | 4.2 | Is the sample representative of the target population? | N | CT | N | Reviewer A: No (authors explicitly state generalizability constrained); Reviewer B: Can't tell (single-program but complete within-program coverage). Final adjudicated rating: No. Authors explicitly acknowledge non-representativeness; single-institution single-year data; institution-specific milestones not generalizable to other IM residencies. Score N consistent with author-acknowledged limitation. Applied consistently across S07/S11/S12 single-program psychometric studies. |
| S11 | Park 2016 | 4.4 | Is the risk of nonresponse bias low? | CT | Y | CT | Reviewer A: Can't tell (substantial submission delays acknowledged, unbalanced ratings); Reviewer B: Yes (complete extraction of submitted evaluations). Final adjudicated rating: Can't tell. Although all submitted evaluations were extracted, the authors explicitly document substantial rater delays (~3 months) and unbalanced numbers of evaluations per resident. Non-response/non-submission is therefore a real issue not directly quantified — score CT (cannot tell whether non-submission introduces bias). |
| S12 | Park 2020 | 4.2 | Is the sample representative of the target population? | N | CT | N | Reviewer A: No (authors explicitly limited to single program); Reviewer B: Can't tell (single-institution sample). Final adjudicated rating: No. Authors explicitly state limited generalizability beyond single program (UI-COM IM residency, n=34). Applied consistently with S07/S11. |
| S12 | Park 2020 | 4.4 | Is the risk of nonresponse bias low? | CT | Y | CT | Reviewer A: Can't tell (substantial unbalance: PGY-2 only ~8 evaluations vs ~27 for PGY-1); Reviewer B: Yes (full cohort followed, mandated assessments). Final adjudicated rating: Can't tell. The Φ reliability dropped to 0.46 in PGY-2 due to substantially lower number of evaluations — unbalanced rater submission introduces a missingness/non-response concern that is acknowledged but not formally analyzed. CT preserves the ambiguity. |
| S14 | Paternotte 2024 | S2 | Do the collected data allow to address the research questions? | CT | Y | CT | Reviewer A: Can't tell (comparative breadth constrained — primarily one trainee's elective experience plus colleague discussions); Reviewer B: Yes (blueprint comparison + EP's elective + reflections map to research questions). Final adjudicated rating: Can't tell. Auto-ethnography with one main auto-ethnographer (EP) plus reflective sessions only partially addresses the comparative research question; data are limited primarily to one trainee's elective experience. Conservatively rated CT. |
| S14 | Paternotte 2024 | 1.4 | Is the interpretation of results sufficiently substantiated by data? | Y | CT | CT | Reviewer A: Yes (extensive blueprint comparison + some quotations); Reviewer B: Can't tell (sparse verbatim data). Final adjudicated rating: Can't tell. While curricular blueprints are documented, interpretive claims about culture and entrustment are sparsely supported with verbatim qualitative data. Conservative scoring per MMAT guidance preserves uncertainty. |
| S14 | Paternotte 2024 | 1.5 | Is there coherence between qualitative data sources, collection, analysis and interpretation? | CT | Y | CT | Reviewer A: Can't tell (mix of personal reflection + blueprint comparison not fully integrated analytically); Reviewer B: Yes (auto-ethnographic framing coherent). Final adjudicated rating: Can't tell. The hybrid of formal blueprint review + personal reflection is partially coherent with auto-ethnographic methodology, but analytic detail and integration between the two strands is limited. Authors themselves acknowledge subjectivity and limited sample. |
| S19 | Schultz 2016 | S1 | Are there clear research questions? | CT | N | CT | Reviewer A: Can't tell (Innovation Report; aims descriptive, not framed as research question); Reviewer B: No (no research question). Final adjudicated rating: Can't tell. As an Innovation Report, the article was not designed as research and lacks a focused research question. Per MMAT guidance, when the criterion is not directly applicable to the design, CT is more appropriate than N. Applied consistently with S15 (also a brief report). |
| S19 | Schultz 2016 | S2 | Do the collected data allow to address the research questions? | CT | N | CT | Reviewer A: Can't tell; Reviewer B: No. Final adjudicated rating: Can't tell. Limited descriptive outcome data (counts of data points, 8/350 extensions, 4 dismissals) only partially address the implementation report's claims; without a formal study design, whether the data "address the research question" cannot be properly judged. |
| S19 | Schultz 2016 | 4.1 | Is the sampling strategy relevant to address the research question? | CT | N | CT | Reviewer A: Can't tell (sampling implicit, whole-program data); Reviewer B: No (no sampling strategy described). Final adjudicated rating: Can't tell. Sampling is implicit (entire program data extraction); appropriate for description but the absence of formal sampling strategy in an Innovation Report does not equate to inappropriate sampling — CT preserves the ambiguity. |
| S19 | Schultz 2016 | 4.3 | Are the measurements appropriate? | CT | N | CT | Reviewer A: Can't tell; Reviewer B: No. Final adjudicated rating: Can't tell. Field notes and EPA-FNs are used as program "data points" but no psychometric measurement is conducted; the article does not present validated outcome measures. Score CT because measurements are not the focus of an Innovation Report. |

# 6. Inter-rater agreement details

| **Statistic** | **Value** | **Notes** |
| --- | --- | --- |
| Number of studies | 20 |  |
| Total items rated by both reviewers | 135 | 7 items per study (S1, S2 + 5 category-specific) for 20 studies; for S18, only S1+S2 are common since reviewers used different categories. |
| Item-level raw agreement (n agreements) | 117 |  |
| Item-level raw agreement (%) | 86.7% |  |
| Cohen's kappa (item-level Y/N/CT) | 0.707 | Unweighted; treats Y/N/CT as nominal categories. |
| % agreement on MMAT category (n=20) | 95.0% (19/20) | 1 disagreement: S18 (Reviewer A: Category 5, mixed methods; Reviewer B: Category 1, qualitative). |
| % agreement on Met/5 totals (n=20) | 85.0% (17/20) |  |
| Mean absolute difference in Met/5 | 0.15 | Average absolute difference between Reviewer A’s and Reviewer B’s Met/5 counts across 20 studies. |

# 7. Methodology

Methodological quality of all 20 included studies was appraised using the Mixed Methods Appraisal Tool (MMAT) version 2018, a single instrument designed to enable critical appraisal of qualitative, quantitative, and mixed methods studies within a single review (Hong et al., 2018). Each study was first screened against two general items (S1: clarity of research questions; S2: whether the collected data allow the research questions to be addressed) and then rated against the five category-specific items appropriate to its design: qualitative (1.1–1.5), quantitative randomised controlled trial (2.1–2.5; not applicable in this review), quantitative non-randomised (3.1–3.5), quantitative descriptive (4.1–4.5), or mixed methods (5.1–5.5). Items were rated Yes, No, or Cannot tell. Two reviewers (A and B) independently appraised every study, including independent design classification, blinded to each other's judgments. Item-level inter-rater agreement was 86.7% (Cohen's κ = 0.707, substantial agreement per Landis & Koch). All disagreements were reconciled by a third reviewer (the adjudicator), who returned to the source PDFs, reviewed the evidence quoted by each reviewer, and made a final rating with quoted justification; one MMAT category disagreement (S18, Ross 2023: A = mixed methods; B = qualitative) was also resolved at the source-document level. We computed Met/5 as the count of Yes ratings on the five category-specific items and applied conservative cut-points of High ≥ 4/5, Moderate = 3/5, and Low ≤ 2/5. We acknowledge that the MMAT developers caution against producing a single overall numeric score and recommend reporting item-level ratings to preserve information about the quality dimensions met (Hong et al., 2018); we therefore retained the full item-level rating table (this Supplementary Material 4) alongside the summary Met/5 score so that readers may inspect the underlying evidence for each item.

Raw per-study scoring files and adjudication notes are available from the corresponding author on request. The MMAT summary, disagreement log, inter-rater agreement statistics, and quality distribution are reproduced in full in this document.
