# Supplementary material for: Programmatic assessment and competency development in postgraduate medical education: a systematic review and narrative synthesis
Source: Front Med (Lausanne). 2026 Jul 16;13:1873126. doi: 10.3389/fmed.2026.1873126 (PMC13422548; doi:10.3389/fmed.2026.1873126)
Supplement: Supplementary file 5 [file Table_5.DOCX]

# Supplementary Material 5

**Programmatic assessment fidelity matrix: mapping of included studies against the four operational features of programmatic assessment**

Studies were mapped against four operational features of programmatic assessment (van der Vleuten et al., 2012; manuscript Section 2.3): (A) longitudinal aggregation of multiple low-stakes data points; (B) triangulation across modalities; (C) formative-first use of individual data points; (D) committee-based synthesis for high-stakes decisions. Ratings: Y, explicitly described; P, partially described; N, absent or contrary; U, unclear (N/U, not described and presence not excludable). The final column gives the synthesis classification. Ratings depend on reporting, so under-described systems may be under-rated (noted in the manuscript’s limitations).

| **Study (system)** | **A** | **B** | **C** | **D** | **Classification** | **Basis (as reported in the source)** |
| --- | --- | --- | --- | --- | --- | --- |
| Acai, 2019 (McMAP) | Y | Y | Y | Y | Designed PA system | Daily WBAs aggregated longitudinally across modules; multiple tools; formative orientation; committee-based decisions described. |
| Ashman, 2025 (AOA21) | Y | Y | P | Y | Designed PA system | 46,180 WBAs aggregated over training; multiple assessment types incl. examinations; formative intent stated but stakeholder data suggest summative experience; committee-based progression decisions. |
| Caretta-Weyer, 2025 (EPA-based CBME) | Y | Y | P | Y | Designed PA system | Programmatic assessment is one of the five implemented core components; EPAs plus complementary data; coaching supports formative use; clinical competency committees synthesize. |
| Chan, 2015 (McMAP) | Y | Y | Y | Y | Designed PA system | Theoretically grounded WBA system; multiple instruments mapped to roles; daily low-stakes assessments; aggregated end-of-rotation synthesis. |
| Gauthier, 2024 (CBD EPA-based WBA) | Y | P | P | Y | Designed PA system | CBD programs aggregate EPA observations longitudinally for competence committee review; study focuses on the WBA component; formative intent present though learner behavior was promotion-driven. |
| Hauff, 2014 (multisource milestone assessment) | N | Y | N | N | Single-time-point multisource assessment | Multi-station, multisource measurement at intern orientation; no longitudinal aggregation, formative program, or committee synthesis described. |
| Lee, 2022 (CBAS e-portfolio) | Y | P | Y | P | Designed PA system | Longitudinal fieldnotes in an e-portfolio; predominantly one instrument type (fieldnotes) from two sources; explicitly formative; program-level review described, committee detail limited. |
| McEwen, 2015 (PASS portfolio) | Y | Y | Y | Y | Designed PA system | Portfolio aggregating field notes and multiple assessment sources; formative emphasis; structured review/decision process. |
| O’Keeffe, 2024 (CTAS) | Y | Y | P | Y | Designed PA system | National multi-assessment system (WBAs, OSCEs, interviews) aggregated for progression decisions; formative orientation less prominent in report. |
| Park, 2016 (NAS end-of-rotation evaluations) | Y | P | N/U | Y | Designed PA system (regulatory NAS context) | Longitudinal aggregation of rotation evaluations feeding clinical competency committee decisions; triangulation and formative-first design thinly described; retained as designed-system evidence with caution. |
| Park, 2020 (Scoring Grid, milestone reporting) | Y | P | N/U | Y | Milestone-based CBME subgroup | Institutional synthesis of evaluations into milestone ratings for ACGME reporting; reporting requirement rather than purposeful PA design; formative use not described. |
| Park, 2021 (ACGME FM milestones, national) | Y | U | N/U | Y | Milestone-based CBME subgroup | National regulatory milestone reporting dataset; committee ratings present; no purposefully designed PA system described. |
| Paternotte, 2024 (CBD / LOGO) | Y | Y | Y | Y | Designed PA systems (two national systems compared) | Both national systems compared are explicitly designed programmatic/portfolio assessment systems; the study is an auto-ethnographic comparison. |
| Perry, 2018 (Master Assessment Plan) | Y | Y | P | P | Designed PA system | Purposeful multi-source assessment mapping; feedback orientation described; committee synthesis less explicit. |
| Rich, 2020 (CBD with EPAs) | Y | Y | Y | Y | Designed PA system | CBD programmatic assessment with EPAs, longitudinal aggregation, formative intent, and competence committees. |
| Rich, 2022 (CBD competence committees) | Y | Y | Y | Y | Designed PA system | Focused on the committee-synthesis feature across four CBD programs. |
| Ross, 2023 (CRAFT) | Y | Y | Y | Y | Designed PA system | National principles-guided programmatic assessment framework with longitudinal low-stakes data, multiple sources, formative orientation, and structured decisions. |
| Schultz, 2016 (EPA-based CBME, 36 EPAs) | Y | Y | P | Y | Designed PA system | Purpose-built competency-based assessment with multiple tools and committee review; formative-first orientation partially described. |
| Schut, 2018 (PA with portfolios) | Y | Y | Y | Y | Designed PA system | All five sampled programs explicitly designed as programmatic assessment with portfolios and mentoring. |
| Woodworth, 2024 (EPA + procedural + NTSA + OSCE) | Y | Y | P | Y | Designed PA system | Purpose-built multi-component system mapped to milestones; piloted across seven programs; committee comparison performed; formative use partially described. |

*Classification reflects whether the source describes a purposefully designed program of assessment; feature ratings are supporting evidence, not a mechanical threshold. Park 2020 and Park 2021, from accreditation-mandated milestone reporting without a described purposeful design, form the milestone-based CBME subgroup. Park 2016 shares their A–D pattern but evaluates a program the institution purposefully structured to feed committee decisions, so it is retained as designed-system evidence (formative-first orientation, feature C, flagged as a caution). Hauff 2014 (N on A, C, and D) is modality evidence, not longitudinal-system evidence.*
