# Supplementary material for: Programmatic assessment and competency development in postgraduate medical education: a systematic review and narrative synthesis
Source: Front Med (Lausanne). 2026 Jul 16;13:1873126. doi: 10.3389/fmed.2026.1873126 (PMC13422548; doi:10.3389/fmed.2026.1873126)
Supplement: Supplementary file 6 [file Table_6.DOCX]

# Supplementary Material 6

**GRADE-CERQual evidence profile: confidence in the principal qualitative and implementation findings**

Confidence in each finding was assessed using GRADE-CERQual (Lewin et al., 2015) across four components: methodological limitations (from the MMAT ratings, Supplementary Material 4), coherence, adequacy, and relevance. Assessments start at High and are downgraded where concerns exist. The quantitative strand was not GRADE-assessed (heterogeneous descriptive designs without comparable effect estimates; manuscript Section 2.9).

| **Summarized review finding** | **Contributing studies (quality rating, Met/5)** | **CERQual confidence** | **Explanation of confidence assessment** |
| --- | --- | --- | --- |
| F1. Learners perceive assessment stakes as a dichotomy rather than a continuum; beyond a threshold, additional low-stakes assessments become a checkbox exercise. | Schut 2018 (High, 5); Gauthier 2024 (High, 5); Acai 2019 (High, 5) | Moderate | Methodological limitations: minor (all contributing studies High). Coherence: good (consistent across settings). Adequacy: moderate (three studies, rich qualitative data). Relevance: moderate concerns (Schut 2018 cohort includes undergraduate learners without stratified reporting; downgraded one level). |
| F2. Faculty are reluctant to document constructive (especially written negative) feedback within the formal system, undermining data quality. | Rich 2020 (High, 5); Ashman 2025 (High, 4); Caretta-Weyer 2025 (Moderate, 3) | Moderate | Methodological limitations: minor-to-moderate (one Moderate-quality study; Ashman survey response rate 18%). Coherence: good (independent convergence across three countries and specialties). Adequacy: moderate. Relevance: direct. Downgraded one level for methodological limitations. |
| F3. Assessment-seeking is driven by promotion requirements rather than learning goals, with avoidance under uncertainty; gaming behaviors occur on both learner and faculty sides. | Gauthier 2024 (High, 5); Acai 2019 (High, 5); Schut 2018 (High, 5) | Moderate | Methodological limitations: minor. Coherence: good. Adequacy: moderate (three studies, two specialty contexts). Relevance: moderate concerns (one study mixes training stages). Downgraded one level. |
| F4. Programmatic assessment requires substantial, sustained resource investment (leadership time, faculty time, software development and maintenance). | Schultz 2016 (Low, 0); Caretta-Weyer 2025 (Moderate, 3); Perry 2018 (Low, 0); McEwen 2015 (Low, 0) | Low | Methodological limitations: serious (three of four contributing studies Low on the descriptive index; descriptive/innovation-report designs). Coherence: good (estimates point the same direction). Adequacy: moderate. Relevance: direct. Downgraded two levels for methodological limitations. |
| F5. Competence committees allocate attention unevenly across resident archetypes, with weakly performing residents consuming disproportionate time and prompting reliance on informal data. | Rich 2022 (High, 5) | Low | Methodological limitations: minor (single High-quality study). Coherence: cannot be assessed across studies (single source). Adequacy: serious concerns (one study, 17 participants, one national context). Relevance: direct. Downgraded two levels for adequacy. |
| F6. Core programmatic assessment constructs (e.g., entrustment) are interpreted differently across national regulatory and cultural contexts. | Paternotte 2024 (Low, 1); supported indirectly by cross-setting variation in Schut 2018 (High, 5) | Low | Methodological limitations: serious (principal source is a Low-quality auto-ethnography). Coherence: plausible and consistent with indirect evidence. Adequacy: serious concerns (essentially one direct study). Relevance: direct. Downgraded two levels. |

*Note. F1–F3 underpin the manuscript’s statements on stakes, feedback documentation, and engagement (Sections 3.3, 4); F4–F6 underpin the resource, committee-functioning, and contextual-variation findings. Confidence: High, Moderate, Low, or Very low, per CERQual.*
